# Fucoidan as a therapeutic agent for ulcerative colitis: mechanisms of action and modulation of the gut microbiota

**DOI:** 10.3389/fcimb.2025.1626614

**Published:** 2025-07-10

**Authors:** Yating Zhang

**Affiliations:** College of Bioscience and Biotechnology, Hunan Agricultural University, Hunan Provincial Engineering Research Center of Applied Microbial Resources Development for Livestock and Poultry, Changsha, Hunan, China

**Keywords:** Fucoidan, ulcerative colitis, gut microbiota, toll-like receptors (TLRs), oxidative stress, inflammation

## Abstract

Ulcerative colitis (UC), a chronic inflammatory bowel disease driven by gut dysbiosis, immune dysregulation, and oxidative stress, lacks universally effective therapies. Fucoidan (FCD), a sulfated polysaccharide derived from brown algae, has emerged as a multifaceted therapeutic candidate due to its anti-inflammatory, antioxidant, and immunomodulatory properties. This review synthesizes FCD’s mechanisms in UC pathogenesis, emphasizing its suppression of NF-κB and MAPK signaling pathways to reduce proinflammatory cytokines (e.g., IL-6, TNF-α) and regulate TLR-mediated macrophage polarization. FCD enhances intestinal barrier integrity via upregulation of tight junction proteins (Claudin-1, ZO-1) and mucin MUC2 expression, while remodeling gut microbial ecology through enrichment of SCFAs-producing bacteria (e.g., Ruminococcaceae) and suppression of pathogens (Escherichia coli, Candida albicans). Preclinical studies highlight LMWF as a superior candidate, demonstrating enhanced bioavailability and efficacy in mitigating DSS-induced colitis. Despite its promise, challenges persist in structural heterogeneity (source- and extraction-dependent), scalable production of LMWF, and insufficient pharmacokinetic data. Emerging strategies—including nanoparticle-based delivery systems and structural modifications (cross-linking, covalent bonding)—aim to overcome bioavailability limitations. This review underscores FCD’s potential as a functional food or adjuvant therapy for UC, while advocating for rigorous clinical validation to bridge translational gaps, Enrichment of SCFAs-producing taxa and suppression of pathobionts (*Escherichia coli*, *Candida albicans*), mediated through prebiotic fermentation. Suppression of NF-κB activation via IκBα stabilization and inhibition of p65 nuclear translocation, and downregulation of MAPK phosphorylation (ERK1/2, JNK, p38), reducing proinflammatory cytokines (IL-6, TNF-α, IL-1β). FCD can be used as a potential treatment for UC.

## 1 Introduction

Inflammatory bowel disease (IBD), which includes ulcerative colitis (UC) and Crohn’s disease (CD), is a chronic, relapsing inflammatory disorder of the gastrointestinal tract. While the specific etiology and pathogenesis of IBD remain elusive, gut microbiota dysbiosis is a hallmark feature. Patients with IBD exhibit reduced microbial diversity compared with healthy individuals, characterized by decreased beneficial taxa such as Lactobacillus spp. and increased abundance of Aspergillus spp. IBD onset spans all age groups, although diagnosis peaks in the third decade of life, particularly in Western industrialized nations. In northern Europe, the annual incidence rates range from 35–50 UC cases and 30–100 CD cases per 100,000 individuals. Global estimates indicate UC burdens of 7.6–245 annual cases and incidence rates of 1.2–20.3 per 100,000 people (The global, regional, and national burden of inflammatory bowel disease in 195 countries and territories, 1990-2017: a systematic analysis for the Global Burden of Disease Study 2017, 2020). Le Berre et al. reported in The Lancet that approximately 5 million people worldwide had UC in 2023 (Le Berre et al., 2023).

Dysbiosis-driven disruption of host–microbiota interactions is central to IBD pathogenesis (Becker et al., 2023). The high inter- and intraindividual variability of the intestinal microbiome influences host susceptibility to immune-mediated disorders, with IBD being particularly sensitive to these perturbations. Immune autophagy plays a pivotal role in maintaining intestinal homeostasis by modulating crosstalk among the gut microbiota, innate/adaptive immunity, and host defense mechanisms (Forbes et al., 2018). Macrophages, key regulators of intestinal inflammation and homeostasis, perform critical functions, including pathogen clearance and dead cell removal. However, in autoimmune disorders, hyperactivated macrophages exacerbate intestinal barrier dysfunction through excessive proinflammatory cytokine production and tissue infiltration (Zhang et al., 2023).

UC is characterized by distal rectal inflammation extending proximally with variable lengths, resulting in a sharp transition between inflamed and noninflamed mucosa. The disease follows a relapsing–remitting clinical course, with over 90% of patients experiencing recurrent flares after initial onset. Early recurrence within the first two years strongly predicts progressive disease severity. While no definitive diagnostic biomarker exists for UC, fecal calprotectin >250 μg/g serves as the most reliable noninvasive marker to screen for IBD, warranting confirmatory endoscopy (Reenaers et al., 2018). Other metalloproteins in the gut microbiota may influence disease progression (Ma Y et al., 2023). However, biomarkers such as fecal lactoferrin (<7.25 μg/g normal threshold) remain insufficiently validated (Ministro and Martins, 2017; Xiao L et al., 2023).

Microbiome profiling of IBD patients with active disease has revealed that intestinal dysbiosis is marked by diminished microbial diversity (particularly within the Firmicutes phylum), reduced community stability, and overrepresentation of Proteobacteria (e.g., *Enterobacteriaceae*) and fungal Ascomycetes relative to healthy controls (Devkota et al., 2012; Gevers et al., 2014; Machiels et al., 2014; Nishino et al., 2018). Dysbiotic shifts correlate directly with intestinal pathology exacerbation. Reactive oxygen species (ROS) serve as key drivers of gut barrier impairment and proinflammatory signaling. Microbiota-derived short-chain fatty acids (SCFAs) reduce the luminal pH, suppressing pathogenic expansion while promoting beneficial taxa to reestablish mucosal homeostasis (Guo et al., 2021). Therapeutic strategies to reconstitute beneficial microbiota include (1) the administration of defined bacterial strains and probiotic formulations that secrete antimicrobial peptides to combat pathogens and (2) enzymatic modulation by probiotics to stimulate symbiotic microbial proliferation, thereby enhancing intestinal homeostasis (Zhou et al., 2019). Probiotics demonstrate multimodal efficacy in IBD management by rectifying dysbiosis, fortifying mucosal barrier integrity, improving microecological niches, and modulating local/systemic immunity—providing a novel therapeutic approach.

Marine seaweed, a vital biological resource, synthesizes diverse bioactive compounds—including polysaccharides, proteins, and polyphenols—with significant pharmaceutical potential. Among these polysaccharides, structurally complex marine polysaccharides have garnered extensive research interest. Functionally, these biopolymers play dual roles: as structural and metabolic reservoirs in marine organisms and as endogenous antioxidants. Algal-derived polysaccharides present across marine flora and fauna exhibit immunomodulatory, anti-inflammatory, antioxidant, and antiviral properties (Smith et al., 2018; Cui et al., 2019; Corino et al., 2021; Jayawardena et al., 2022). Preclinical studies have demonstrated that seaweed polysaccharides and extracts protect the gastric mucosal barrier and attenuate inflammation, highlighting their therapeutic potential for gastrointestinal disorders (Lajili et al., 2019; Wang et al., 2019; Li R. et al., 2024). Notably, algal polysaccharides resist degradation by host digestive enzymes and act as prebiotic substrates for probiotic fermentation, positioning them as promising candidates for managing intestinal inflammatory diseases such as IBD (Xie et al., 2019).

## 2 Physiological processes and factors affecting Fucoidan absorption and metabolism

### 2.1 Brief description of FCD

Marine macroalgae (seaweeds), among the ocean’s most biologically valuable resources, are taxonomically classified into three groups—brown (Phaeophyceae), red (Rhodophyta), and green (Chlorophyta) algae—on the basis of pigmentation, monosaccharide profiles, molecular weight distributions, and structural polysaccharide composition (Xu et al., 2017). Fucoidan (FCD), a sulfated polysaccharide predominantly abundant in brown algae, coexists with other characteristic biopolymers, such as alginates and laminarin. FCD-producing species span diverse marine taxa, including *Laminaria hyperborea*, *Sargassum stenophyllum*, *Hizikia fusiforme*, *Ascophyllum nodosum*, *Fucus* spp. (*F. evanescens*, *F. serratus*, *F. distichus*, *F. vesiculosus*), *Analipus japonicus*, *Caulerpa racemosa*, *Chorda filum*, *Padina gymnospora*, *Kjellmaniella crassifolia*, and *Dictyota menstrualis*.

FCD, a sulfated polysaccharide with the molecular formula (C_6_H_10_O_7_S)_n_, contains sulfate groups as integral components of its structural composition. Predominantly isolated from brown algae, this biopolymer consists primarily of sulfated fucose residues (Koh et al., 2019), with minor constituents, including xylose, mannose, galactose, rhamnose, arabinose, glucose, glucuronic acid, and acetyl groups (Jin et al., 2019; Koh et al., 2019; Usoltseva et al., 2019). Located in the cell walls of brown algae, FCD contributes to structural integrity by preventing tissue dehydration and stabilizing cell membranes (Lakshmana Senthil, 2024).

**Figure 1:**
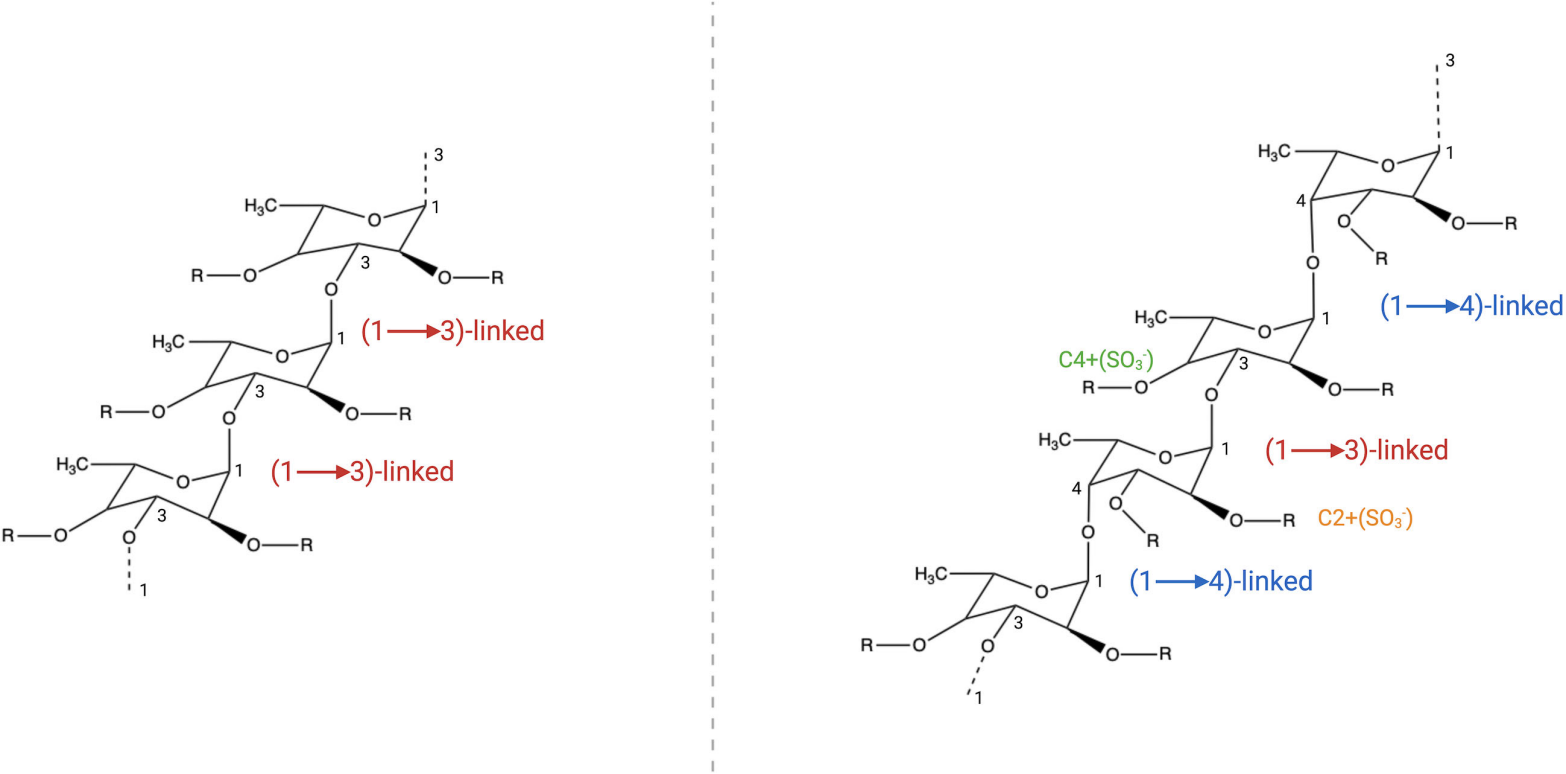
FCD structural diversity. Two predominant backbone motifs are observed (1): a 1,3-linked α-L-fucopyranose backbone and (2) a heteroglycosidic framework with alternating 1,3- and 1,4-linked α-L-fucopyranose residues. The C-2 and C-4 positions of these fucose residues are susceptible to sulfation (SO_3_^-^ substitution), contributing to structural variability.

Structurally, FCD exhibit species-specific variations. Two common α-L-fucopyranose backbone motifs are observed (1): a 1,3-linked framework and (2) alternating 1,3- and 1,4-linked residues. Sulfation typically occurs at the C-2 or C-4 positions of the fucose residues (**Figure 1**) (Jia et al., 2025). While brown algal FCD share conserved backbone structures, they diverge in their sulfation patterns and glucuronic acid contents. For example, *Fucus serratus* (silky brown algae), *Fucus distichus* (pteridophyte brown algae), and *Pelvetia canaliculata* (tubular brown algae) exhibit similar core skeletons but differ in their branching complexity and monosaccharide diversity, which are correlated with functional distinctions (Ale et al., 2011; Wang et al., 2021; Obluchinskaya et al., 2023). Notably, certain FCD (e.g., from *Bifurcaria* and *Himanthalia elongata*) deviate from these canonical frameworks, suggesting additional structural heterogeneity (Sanjeewa et al., 2017).

The sulfate groups of FCD confer distinct biological activities. Studies have demonstrated its ability to mitigate intestinal barrier dysfunction through three mechanisms (1): enhancing tight junctions (TJs) and adherens junctions (AJs) protein interactions in epithelial cells (2), restoring the gut microbiota composition, and (3) suppressing proinflammatory cytokine production (Iraha et al., 2013; Shi et al., 2017; Luo et al., 2021). Both fucose-oligomeric polyphenol conjugates and desulfated FCD—structurally distinct precursors—alleviate dextran sulfate sodium (DSS)-induced acute colitis by suppressing inflammatory cytokine signaling (Lean et al., 2015). FCD has also been extensively studied for its antitumor, anticoagulant, and antioxidant activities; ability to modulate glucose and cholesterol metabolism; and potential hepatoprotective and nephroprotective effects.

The key bioactive properties of FCD are primarily attributed to its sulfated polysaccharide structure. Due to its complex structure, FCD cannot be predicted or classified. FCD exerts significant immunomodulatory, antitumor, and anti-inflammatory effects. Notably, FCD demonstrates pronounced impacts on intestinal function. An *in vitro* study by Chen et al. demonstrated that FCD modulates gut microbiota composition by altering SCFAs production (Chen et al., 2023). Furthermore, in a DSS-induced IBD model, Ye et al. showed that FCD intervention restructured gut microbial communities, enhanced colonic barrier integrity through upregulation of tight junction proteins (e.g., claudin-1, occludin), and suppressed colonic inflammation via the TLR4/NF-κB signaling pathway (Ye et al., 2024). Collectively, these findings highlight the therapeutic potential of FCD in IBD management.

### 2.2 Pharmacokinetics of FCD

Preclinical studies have characterized the absorption, distribution, metabolism, and excretion (ADME) properties of FCD. Absorption is quantified via enzyme-linked immunosorbent assay (ELISA) using FCD-specific antibodies (Torode et al., 2015; Pozharitskaya et al., 2018). In rats, oral administration of 737 kDa FCD resulted in peak serum concentrations at 4 hours postadministration, with renal deposition observed in nephrons (Nagamine et al., 2014). Systemic accumulation in extrarenal organs has also been documented (Nagamine et al., 2014). Notably, low-molecular-weight FCD (LMWF) has enhanced therapeutic potential; comparative pharmacokinetic analyses between LMWF and medium-molecular-weight FCD (MMWF) have shown superior bioavailability and tissue uptake of LMWF, supporting its clinical applicability. While FCD exhibits a favorable pharmacokinetic profile with minimal toxicity, inadequate data exist regarding its systemic redistribution.

A recent study by Kadena et al (Kadena et al., 2018). evaluated FCD absorption via oral administration. The authors proposed that dietary seaweed consumption influences FCD uptake. In their trial, 396 participants consumed up to 3 g of *Cladosiphon okamuranus* (Okinawan Mozuku), with urinary FCD detected in 385 individuals. In addition, Ikeda-Ohtsubo et al (Ikeda-Ohtsubo et al., 2020). investigated the *in vitro* modulation of the gut microbiota by FCD. High-purity (>95%) brown seaweed FCD (49.8 kDa), which was isolated from C. okamuranus cultivated in Japan, was administered to adult zebrafish at a 1:1 (w/w) feed ratio for 21 days. Pro- and anti-inflammatory cytokine levels were quantified via quantitative PCR (qPCR), while microbiota shifts were assessed by 16S rRNA sequencing. Compared with control zebrafish, FCD-supplemented zebrafish presented significantly reduced IL-1β (proinflammatory) expression. Additionally, FCD enhanced intestinal microbial diversity and altered community composition, favoring beneficial taxa.

While additional investigations are needed to elucidate the mechanisms of intestinal FCD absorption, multidisciplinary approaches remain critical to comprehensively characterize its pharmacokinetic behavior. The systematic accumulation of these data will establish a robust foundation for the translation of FCD into clinically viable therapeutics.

## 3 The gut microbiota and UC

Short-chain fatty acids (SCFAs), metabolites generated by the gut microbiota, reduce the intestinal pH (Jiang MZ et al., 2024), suppress pathogenic bacterial growth, and promote beneficial taxa, thereby stabilizing microbial communities (Guo et al., 2021). Dysbiosis triggers gut inflammation by enabling harmful bacterial overgrowth. This imbalance increases enterotoxin release, which increases mucosal permeability and immunosuppressive protein production, ultimately disrupting immune homeostasis. Subsequent antigen-presenting cell activation drives T-cell subset polarization toward proinflammatory phenotypes, exacerbating tissue damage (Tang et al., 2019).

The gut microbiota sustains host homeostasis by regulating metabolic processes, epithelial barrier integrity, and immune system development and function (Luthuli et al., 2019; Sittipo et al., 2019). Gut microbiota-derived metabolites, such as SCFAs (e.g., butyrate, propionate, acetate), circulate systemically to organs, including the gut, spleen, liver, and pancreas. These metabolites regulate gastrointestinal hormone secretion, modulate blood glucose and lipid metabolism, and attenuate insulin resistance and inflammation, exerting broad physiological and immunomodulatory effects (Schoeler and Caesar, 2019; Liu et al., 2022). SCFAs additionally activate intestinal epithelial cells, reduce susceptibility to inflammatory disorders, and fine-tune innate and adaptive immune responses.

Commensal gut microbes competitively inhibit pathogenic bacteria via the production of antimicrobial compounds (Peng et al., 2024), such as bacteriocins and hydrogen peroxide. Coevolution with the host immune system has enabled sophisticated discrimination between the commensal microbiota and pathogens, preserving antimicrobial defense capacity. The microbiota critically shapes both innate and adaptive immune response maturation and activity (Matson et al., 2021).

Patients with inflammatory bowel disease (IBD) exhibit a dysbiotic gut microbiota marked by increased abundances of *Mycobacterium avium* (Naser et al., 2014) and *Escherichia coli* (Cho et al., 2022), alongside diminished levels of butyrate and other SCFAs (Akhtar et al., 2022). These SCFAs suppress histone deacetylase (HDAC) activity (Fawad et al., 2022), modulate gene expression and cellular proliferation, and regulate immune responses. Notably, adherent-invasive *E. coli* strains demonstrate enhanced mucosal adhesion and invasion, triggering pathogenic immune activation. Emerging evidence highlights microbial metabolites—particularly SCFAs such as butyrate—as critical mediators of gastrointestinal barrier integrity and mucosal homeostasis rather than microbiota composition alone.

Butyrate serves as the primary energy source for colonic epithelial cells, promoting stem cell proliferation and anti-inflammatory macrophage polarization via HDAC inhibition and histone acetyltransferase activation. Conversely, butyrate depletion exacerbates colonic inflammation by impairing regulatory T (Treg) cell differentiation and reducing macrophage antimicrobial activity. The severity of microbial dysbiosis is directly correlated with IBD progression.

Patients with active IBD display reduced microbial diversity compared with non-IBD controls. Proteobacteria levels are significantly elevated during active UC (UC) but decline during remission. Active UC is further associated with increased abundances of *Klebsiella*, *Enterococcus*, and *Haemophilus*, whereas remission correlates with enrichment of *Roseburia*, *Faecalibacterium*, *Bradyrhizobium*, and *Enterococcus faecalis*.

### 3.1 Escherichia coli

**Figure 2:**
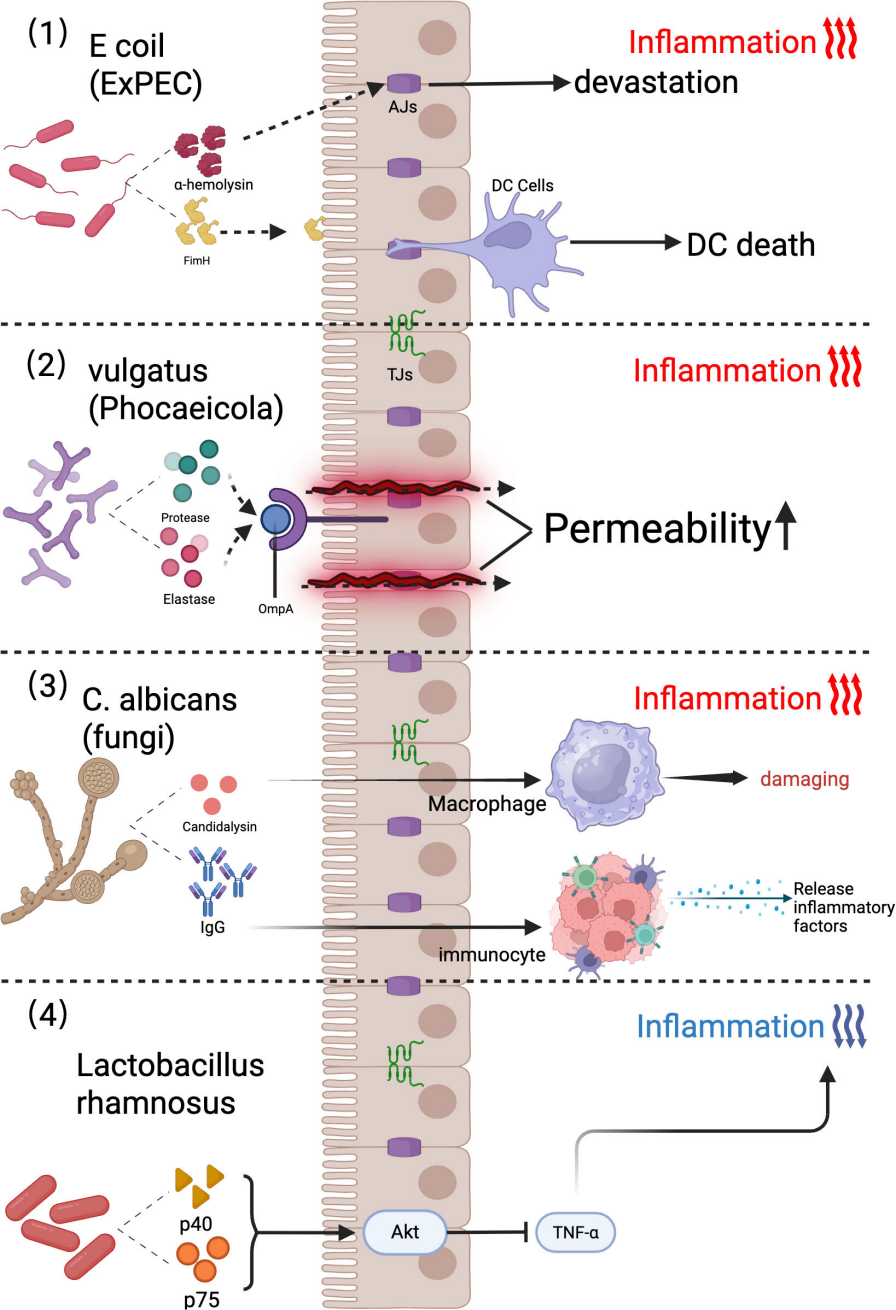
Mechanisms of gut microbiota in promoting and suppressing ulcerative colitis. (1) *Escherichia coli* strains harboring ExPEC virulence genes, such as FimH (mediating intestinal epithelial cell [IEC] adhesion) and α-hemolysin (disrupting IEC tight junctions), promote epithelial barrier dysfunction, DC infection, and host cell death. (2) The gut commensal bacterium *Phocaeicola vulgatus* (formerly *Bacteroides vulgatus*) acts as a patient-specific biomarker, secreting proteases and elastases that increase intestinal permeability and correlate with UC severity. (3) Mechanisms Underlying *Candida albicans*-Induced Inflammation. (4) The probiotic *Lactobacillus rhamnosus* attenuates inflammation by secreting the p40 and p75 proteins, which activate the Akt signaling pathway to suppress TNF-α-induced epithelial apoptosis in human colonic epithelia and murine models. ExPEC, Extraintestinal Pathogenic; OmpA, Outer Membrane Protein A; DC Cells, Dendritic Cells; Akt, Ak strain transforming; TNF-α, Tumor Necrosis Factor alpha; AJs, Adherens Junctions; TJs, Tight Junctions; FimH, Fimbrial tip adhesin subunit H; IgG, Immunoglobulin G.

UC-associated pathogens include diverse *Escherichia coli* strains, such as diffuse adherent *E. coli* (DAEC) (Walczuk et al., 2019) and extraintestinal pathogenic *E. coli* (ExPEC) (**Figure 2**). In 1987, Burke and Axon demonstrated that fecal isolates from UC patients predominantly comprised DAEC strains exhibiting enterotoxigenic and enteropathogenic traits, unlike those from healthy individuals. ExPEC strains harbor virulence genes encoding toxins (e.g., α-hemolysin) and adhesins (e.g., FimH) (**Figure 2**) (Mirsepasi-Lauridsen et al., 2016; Mirsepasi-Lauridsen et al., 2019). Unlike pathogens that target small intestinal epithelia, these strains produce hemolysins that compromise intestinal epithelial cell (IEC) tight junction integrity, exacerbating colitis in murine models (Mirsepasi-Lauridsen et al., 2016; Mirsepasi-Lauridsen et al., 2020; Yang et al., 2020).

### 3.2 Bacteroides vulgatus

The genus *Bacteroides*, particularly *Bacteroides vulgatus*, is enriched in the colonic submucosal microbiota of UC patients. Notably, elevated serum titers of OmpA antibodies targeting *Brucella* outer membrane proteins are observed in a subset of individuals with UC (**Figure 2**) (Sharma et al., 2022).

### 3.3 *Candida albicans* (fungi)

*Candida albicans* is a key fungal commensal in the human gut microbiota that contributes to immune system development through mechanisms such as systemic antifungal IgG production (**Figure 2**) (Doron et al., 2021; Shao et al., 2022a). However, this fungus exacerbates intestinal inflammation via a self-reinforcing cycle: low-grade gut inflammation promotes fungal colonization, whereas *C. albicans* overgrowth amplifies inflammatory responses. Inflammation-driven *C. albicans* proliferation worsens colitis by lysing macrophages and secreting candidalysin, a hyphal exotoxin. Candidalysin directly induces intestinal inflammation, and *in vitro* studies have demonstrated that IL-1β production is correlated with UC severity (Doron et al., 2021; Shao et al., 2022a).

Systemic antifungal IgG production depends on the CARD9 signaling pathway (**Figure 2**) (Doron et al., 2021). The DSS-induced murine colitis model recapitulates the clinical and histopathological features of human UC (Yang and Merlin, 2024). *Card9*-deficient mice exhibit impaired immune responses, including reduced expression of IL-6, IL-17A, and IL-22 and regenerating islet-derived protein 3γ (RegIIIγ) after DSS challenge (Sokol et al., 2013).

*Candida albicans*, a pathobiont of the gut microbiota, exacerbates UC via mycelium-associated hemolysin production (Moyes et al., 2016; Li et al., 2022). Strain-dependent variations in *C. albicans* macrophage lytic activity further modulate disease progression (**Figure 2**) (Li et al., 2022). While *C. albicans* has been implicated in the severity of Crohn’s disease, recent studies have associated its abundance with chronic prostatitis progression, with increased colonization in advanced cases (Li et al., 2022; Hidalgo-Vico et al., 2024).

Studies have demonstrated the critical role of yeast morphology in *Candida albicans* adaptation (1): Deletion of EFG1 (Enhanced Filamentous Growth Protein 1) (Park et al., 2020) or FLO8 (Flocculation Protein 8, key regulators of filamentation) (Hidalgo-Vico et al., 2024) enhances fungal colonization in the antibiotic-treated murine gut (2). Heterologous expression of UME6 (Transcriptional Repressor UME6, a hyphal transition driver) (Shao et al., 2022b) reduces colonization capacity (3). ZCF8 (Zinc Finger Transcription Factor UME6-interacting Protein 2), TRY4 (Trypsin-4), and ZFU2 (Zinc Finger Transcription Factor UME6-interacting Protein 2) —negative regulators of filamentation—are essential for fungal survival in abiotic environments (Böhm et al., 2017) (4). Null mutations in HMS1 (Hyphal Morphology and Stress Response Protein 1) (Wakade et al., 2023) and CPH2 (cAMP-dependent Protein Kinase Homolog 2, yeast morphology promoters under anaerobic conditions) impair colonization (5). Yeast morphology is dominant in murine gut symbionts, suggesting fitness for intestinal niches (Zhang et al., 2025).

While yeast forms thrive in the gut, filamentous morphotypes may colonize other mucosae. For example, hyphal structures are prevalent in the oral mucosa (Jin et al., 2019), although their presence does not inherently indicate pathogenicity.

### 3.4 Probiotics (Lactobacilli)

Probiotic efficacy requires survival in the gastrointestinal (GI) tract, gastric acid tolerance, the absence of antibiotic resistance genes, and demonstrable host benefits (Nya, 2022). Among probiotic candidates, *Lactobacillus* spp. remain the most widely utilized and studied (**Figure 2**).

Lactobacilli modulate GI immune responses by enhancing intestinal barrier integrity and inhibiting pathogen colonization (Kong et al., 2020). These effects are mediated through interactions with innate and adaptive immune cells via microbe-associated molecular patterns (MAMPs), which engage pattern recognition receptors (PRRs), such as Toll-like receptors (TLRs), nucleotide-binding oligomerization domain (NOD)-like receptors, and C-type lectins (Veiga et al., 2010). Additionally, Lactobacillus species secrete immunoregulatory proteins. For example, *Lactobacillus rhamnosus* GG (ATCC 53103) secretes p40 and p75 proteins, which activate the Akt signaling pathway and inhibit TNF-α-induced epithelial cell apoptosis in human and murine colonic epithelial cells (**Figure 2**).

Synbiotic approaches combining Bifidobacterium lactis with lactobacilli reduce colitis severity and Enterobacteriaceae levels—a family associated with colitogenic potential—during early disease stages (Veiga et al., 2010). Many *Lactobacillus* species negatively correlate with UC severity. DeMarco et al (De Marco et al., 2018). demonstrated that the probiotic strains *Lactobacillus acidophilus*, L. casei, *Lactococcus lactis*, L. reuteri, and Saccharomyces boulardii exert anti-inflammatory effects by selectively modulating the production of cytokines—including IL-10, IL-1β, TNF-α, PGE-2, and IL-8—in HT-29 intestinal epithelial cells. Jia et al. alleviated colitis by increasing intestinal macrophage proportions and IL-10 secretion via *Lactobacillus johnsonii* (Jia et al., 2022). Sun et al. attenuated DSS-induced UC through *L. plantarum*-12 administration, enhancing barrier function by upregulating Mucin 2 (MUC2) protein expression (Sun et al., 2020).

Collectively, these findings demonstrate that probiotics attenuate inflammation by suppressing proinflammatory cytokines (e.g., IL-1β and TNF-α) and enhancing anti-inflammatory mediators (e.g., IL-10) through PRR- and Akt-dependent pathways.

## 4 Inflammation in UC

UC is characterized by intestinal barrier dysfunction, which originates from epithelial cell impairment or structural defects in the intestinal epithelium. Dysregulated immune mediators and aberrant cellular activity in the lamina propria further compromise barrier integrity, triggering a cascade of inflammation that drives disease chronicity.

**Figure 3:**
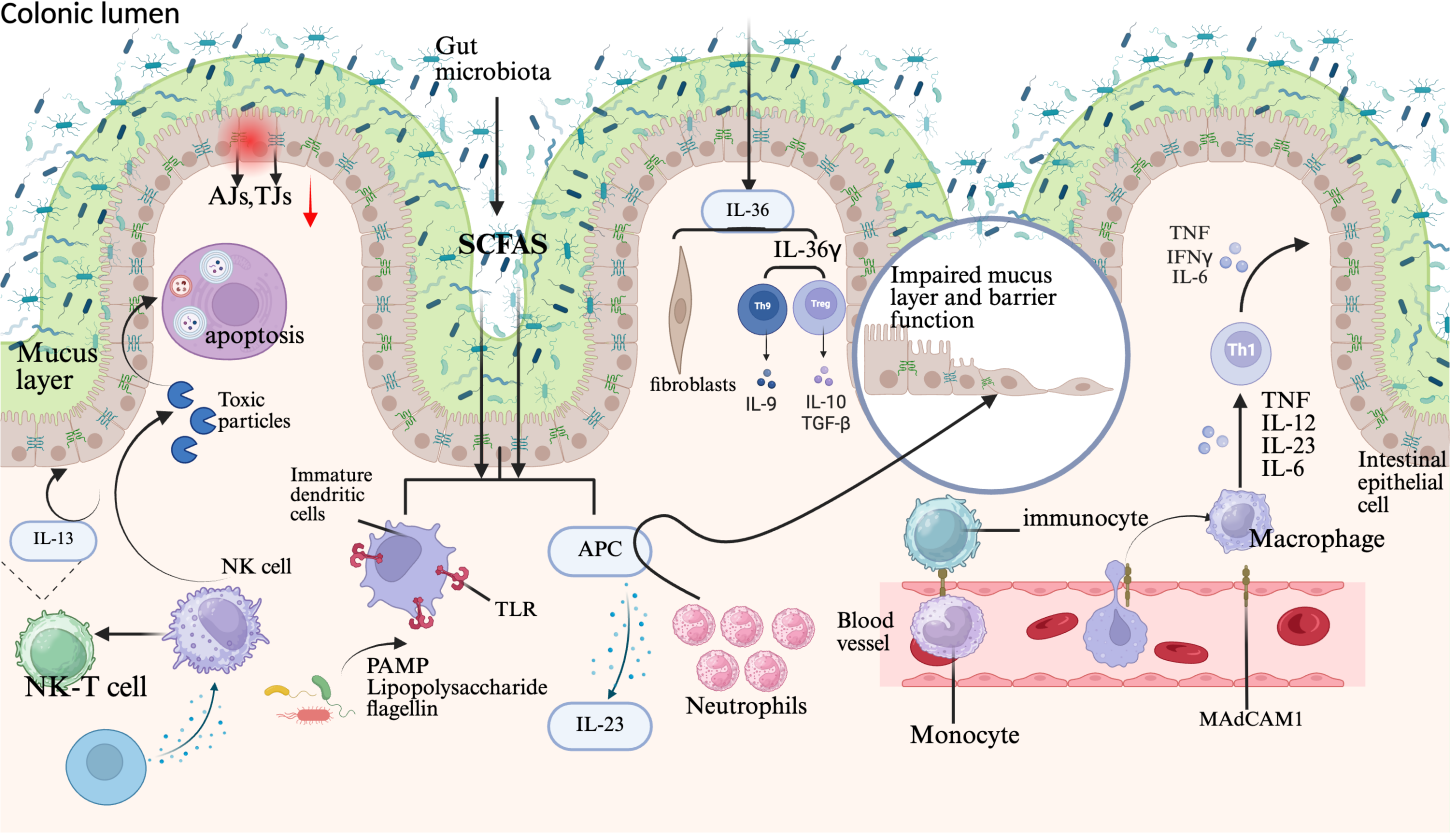
Pathophysiology of UC. UC pathophysiology involves multifactorial contributors, including reduced intestinal microbiota diversity and metabolic alterations such as diminished SCFAs production. In UC patients, the colonic mucus layer is compromised due to decreased synthesis of MUC2. These microbiota shifts and mucus layer thinning disrupt intestinal barrier integrity, facilitating microbial translocation to the epithelial barrier. Focal apoptosis and dysregulated TJs protein expression further degrade the intestinal epithelium, enabling microbiota penetration. This breach activates macrophages and APCs, triggering chemokine production and neutrophil recruitment. Neutrophils mediate primary cellular defense via neutrophil extracellular trap (NET) formation, whereas immune cell infiltration occurs through vascular epithelium-expressed adhesion molecules. Infiltrating monocytes differentiate into macrophages that secrete TNF, IL-12, IL-23, and IL-6, driving TH1 cell polarization. Concurrently, epithelial-derived IL-36γ suppresses Treg cells and promotes IL-9-producing TH9 cell polarization, whereas IL-36 activates fibrogenic genes. Additionally, IL-13 released by NKT cells exacerbates barrier dysfunction. AJs, Adherens junctions; TJs, Tight junctions; SCFAs, Short-chain fatty acids; NK cell, Natural killer cell; NK-T cell, Natural killer T cell; MAdCAM-1, Mucosal vascular addressin cell adhesion molecule 1; TLR, Toll-like receptor; IFNγ, Interferon gamma; TNF, Tumor necrosis factor; IL-12, Interleukin-12; IL-23, Interleukin-23; IL-6, Interleukin-6; IL-9, Interleukin-9; IL-10, Interleukin-10; TGF-β, Transforming growth factor beta; IL-13, Interleukin-13; APC, Antigen-presenting cell; IL-36, Interleukin-36; IL-36γ, Interleukin-36 gamma.

An extensive network of dendritic cells resides beneath the intestinal epithelium. When substantial quantities of Aspergillus bacteria breach the mucus barrier, pathogen-associated molecular patterns (PAMPs), such as lipopolysaccharides and flagellin, on the fungal surface are detected by TLRs on dendritic cells (**Figure 3**) (Kawai and Akira, 2011; Wells et al., 2011). Immature dendritic cells secrete IL-23, driving localized intestinal inflammation (Dewayani et al., 2021). Cytokine-activated natural killer (NK) cells adhere to affected vascular smooth muscle cells (VSMCs), releasing cytotoxic granules that induce apoptosis. IL-1β further stimulates innate lymphoid cells (ILCs) to produce IFN-γ, IL-17, and other proinflammatory cytokines, amplifying the inflammatory cascade (**Figure 3**) (Bishop et al., 2014).

The progression of UC is driven by dysregulated pro- and anti-inflammatory chemokine signaling networks. Oxidative stress exacerbates intestinal inflammation in UC by activating nuclear factor erythroid 2-related factor 2 (Nrf2), which triggers nuclear translocation to induce antioxidant response pathways (**Figure 3**). Furthermore, Nrf2 enhances mucosal barrier integrity through the transcriptional regulation of intestinal tight junction proteins, thereby suppressing UC pathogenesis.

## 5 FCD and UC

FCD exerts multifaceted effects across the inflammatory cascade in UC. These include (1) inhibition of lymphocyte adhesion and infiltration (2); suppression of enzymatic activity, notably matrix metalloproteinases (MMPs) and the complement cascade (3); modulation of inflammation-associated gene expression and transcriptional regulation; and (4) induction of apoptotic cell death. FCD also effectively prevents inflammatory lesion formation *in vitro* (Pomin, 2015; Apostolova et al., 2020; Ahmad et al., 2021) and influences critical therapeutic targets implicated in the pathogenesis and progression of inflammatory disorders (Wu et al., 2022).

The anti-inflammatory properties of FCD have been extensively investigated, primarily through *in vitro* studies using murine RAW 264.7 macrophages. Liyanage et al (Liyanage et al., 2023). demonstrated that FCD suppresses lipopolysaccharide (LPS)-induced prostaglandin E2 (PGE2) production in these cells, highlighting its central role in mediating anti-inflammatory effects. Additionally, FCD prevented the nuclear translocation of NF-κB p65, stabilized IκBα against degradation, and inhibited the MAPK signaling pathway. In Murraya-derived FCD, reduced secretion of TNF-α and IL-1β, along with suppressed neutrophil infiltration, further underscored its efficacy in attenuating early-phase inflammation (Liyanage et al., 2023).

The intestinal barrier is structurally defined by intestinal epithelial cells (IECs) interconnected via AJs, TJs, and associated complexes (Bhat et al., 2018). TJs interface with the cytoskeletal framework, forming a dynamic barrier system. FCD enhances gut barrier integrity by (1) increasing transmembrane protein activity (2), activating TJ-associated signaling pathways, and (3) reducing paracellular permeability in IECs. Intestinal PRRs, including TLRs and nucleotide-binding oligomerization domain-like receptors (NLRs), bind FCD and trigger signaling cascades that modulate inflammatory cytokines and immune mediators. These interactions culminate in upregulated expression of barrier-protective genes and proteins, reinforcing intestinal immunity (Bhat et al., 2018).

FCD fermentation in the colon enhances microbial diversity, increases SCFAs production (e.g., acetate, succinate), and mitigates colitis severity in murine models (Fan LJ et al., 2023). Specifically, FCD supplementation elevates fecal acetate and succinate levels in colitis-prone mice, counteracting antibiotic-induced dysbiosis (Wang et al., 2020). *Arthrospira*-derived FCD increases the abundance of Lactobacillus, a genus critical for intestinal immunity and epithelial renewal (Shang et al., 2016; Lee et al., 2018). Dietary FCD also enriches *Ruminococcaceae*, the primary producers of SCFAs that modulate the Th1/Treg cell balance to maintain gut homeostasis (Smith et al., 2013; Sun et al., 2018). Additionally, the alga FCD strengthens mucosal immunity by increasing mucin and secretory IgA production in the intestinal lumen (Takahashi et al., 2020). Collectively, these findings position dietary FCD as a potent modulator of microbiota composition, with broad implications for gastrointestinal health (Li R. et al., 2024; Li S. et al., 2024).

FCD modulates the composition of the gut microbiota by enhancing intestinal probiotic populations and stimulating epithelial cells to secrete SCFAs, antimicrobial peptides (AMPs), and MUC2—a gel-forming mucin critical for the colonic and small intestinal mucus layers. These IEC secretory complexes inhibit bacterial adhesion in the intestinal lumen (Sicard et al., 2017; Paone and Cani, 2020). Conversely, increased intestinal permeability (a risk factor for bacterial translocation and disease pathogenesis) is counteracted by the reinforcement of the intestinal chemical barrier by FCD. Furthermore, FCD induces the synthesis of autoimmune mediators, including secretory immunoglobulin A (sIgA), which is produced by lamina propria plasma cells and serves as the predominant immunoglobulin in intestinal secretions. sIgA prevents pathogen invasion and strengthens the microbial barrier, thereby strengthening gut immune homeostasis.Notably, FCD from Acaudina molpadioides ameliorated cyclophosphamide (CPA)-induced mucosal damage by reducing intestinal inflammation, increasing TJ protein expression, and enriching SCFAs-producing microbiota (*Coprococcus*, *Rikenella*, and *Butyricicoccus*).

### 5.1 NF-κB pathway

**Figure 4:**
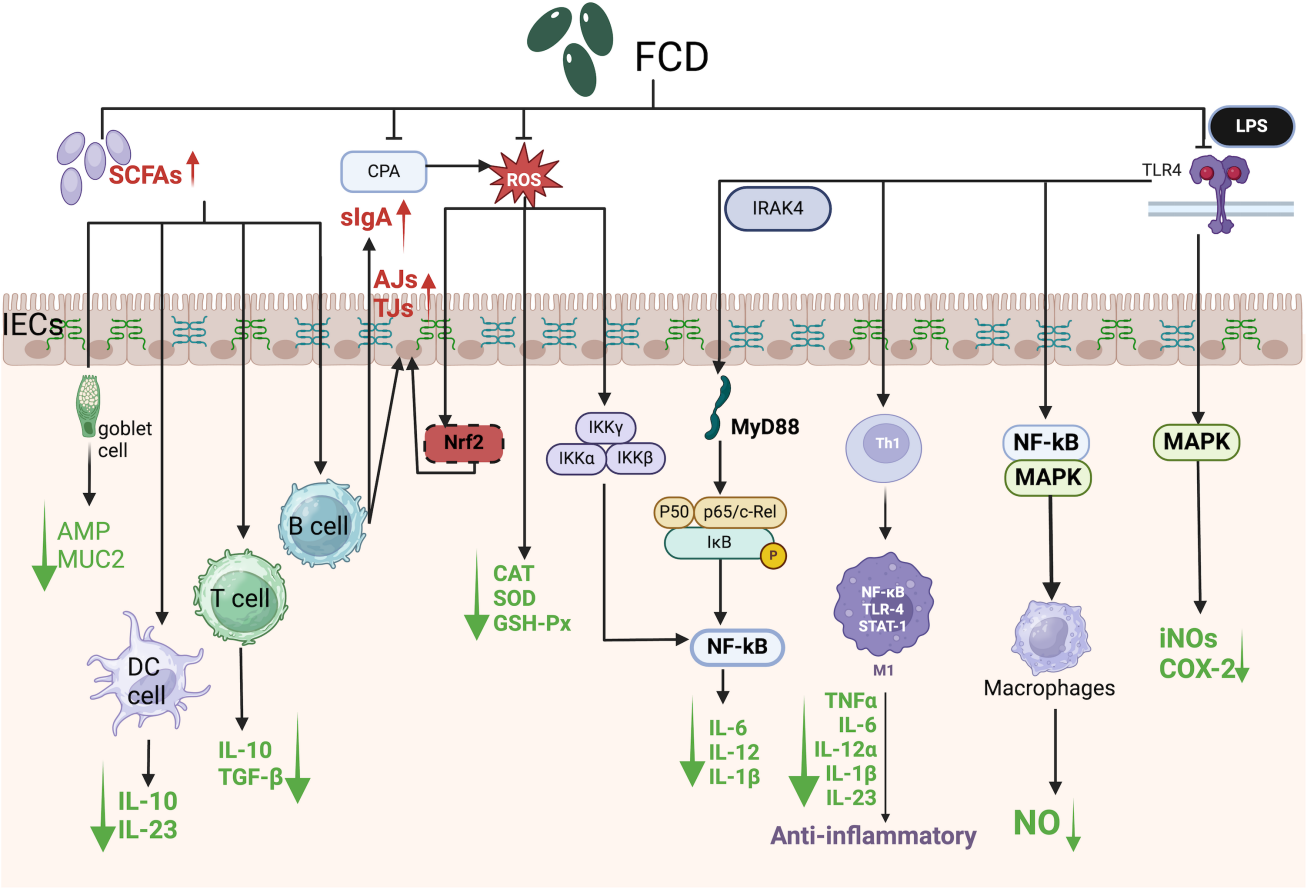
Schematic of signaling pathways mediating the protective effects of algal polysaccharides against intestinal damage. SCFAs, Short-chain fatty acids; CPA, Cyclophosphamide; ROS, Reactive oxygen species; LPS, Lipopolysaccharide; TLR4, Toll-like receptor 4; slgA, Secretory immunoglobulin A; IRAK4, Interleukin-1 receptor-associated kinase 4; IECs, Intestinal epithelial cells; AJs, Adherens junctions; TJs, Tight junctions; AMP, Antimicrobial peptide; MUC2, Mucin 2; DC cell, Dendritic cell; T cell, T lymphocyte; B cell, B lymphocyte; Nrf2, Nuclear factor erythroid 2-related factor 2; IKKα, Inhibitor of nuclear factor kappa-B kinase subunit alpha; IKKβ, Inhibitor of nuclear factor kappa-B kinase subunit beta; IKKγ, Inhibitor of nuclear factor kappa-B kinase subunit gamma; MyD88, Myeloid differentiation primary response 88; IκB, Inhibitor of kappa B; Th1, T helper 1 cell; M1, Classically activated macrophage; STAT-1, Signal transducer and activator of transcription 1; IL-10, Interleukin-10; IL-23, Interleukin-23; TGF-β, Transforming growth factor beta; CAT, Catalase; SOD, Superoxide dismutase; GSH-Px, Glutathione peroxidase; IL-6, Interleukin-6; IL-12, Interleukin-12; IL-1β, Interleukin-1 beta; TNFα, Tumor necrosis factor alpha; IL-12α, Interleukin-12 subunit alpha; iNOS, Inducible nitric oxide synthase; COX-2, Cyclooxygenase-2; NO, nitric oxide.

The NF-κB transcription factor exists as homo or heterodimeric complexes (e.g., p50/p65) bound to the inhibitory protein IκBα under basal conditions. Upon stimulation, IκBα is phosphorylated and ubiquitinated, leading to proteasomal degradation and subsequent release of NF-κB subunits (e.g., p65), which translocate to the nucleus to activate proinflammatory target genes (**Figure 4**) (Hu et al., 2018). In a murine chronic colitis model, Cladosiphon okamuranus Tokida-derived FCD inhibited NF-κB pathway activation, reducing IL-6 and IFN-γ levels and ameliorating disease severity (Matsumoto et al., 2004). This compound also suppressed LPS-induced secretion of nitric oxide (NO), prostaglandin E2 (PGE2), TNF-α, and IL-1β in macrophages without inducing cytotoxicity. Notably, low-dose FCD enhanced the production of NO, inducible nitric oxide synthase (iNOS), ROS, and cytokines (IL-1β, IL-6, IL-12, and TNF-α) by macrophages, whereas high-dose FCD attenuated these mediators, revealing a concentration-dependent biphasic anti-inflammatory response.

NF-κB signaling is central to inflammatory pathogenesis (Zahan et al., 2022). FCD attenuated the LPS-induced nuclear translocation of NF-κB p65 and the accumulation of intracellular ROS in RAW 264.7 macrophages (**Figure 4**). Furthermore, it reversed CPA-induced immunosuppression by reducing ROS levels and downregulating proinflammatory cytokines (IL-6, IL-1β, and IL-12) via NF-κB pathway inhibition, thereby restoring intestinal immune homeostasis (Zahan et al., 2022).

Mounting evidence demonstrates FCD ‘s inhibitory effects on NF-κB signaling. K.K. Asanka Sanjeewa et al. isolated FCD from *Padina commersonii* and stimulated RAW264.7 cells, revealing that FCD significantly downregulates mRNA and protein expression of TLR2, TLR4, and MyD88 in LPS-induced inflammation, thereby suppressing inflammatory responses (Asanka Sanjeewa et al., 2019). In an *in vivo* model of UC induced by a fiber-deficient diet, Weiyun Zheng et al. found that FCD inhibits NF-κB pathway activation, reduces intestinal LPS levels, ameliorates inflammatory cell infiltration, and preserves gut barrier integrity, collectively mitigating UC pathology (Zheng et al., 2024).

### 5.2 Mitogen-activated protein kinase pathway

The MAPK family comprises phosphotransferases that regulate cytosolic signaling cascades via the phosphorylation of serine/threonine residues (Braicu et al., 2019). Researchers have quantified the expression of three MAPK isoforms: extracellular signal-regulated kinase 1/2 (ERK1/2), phosphorylated c-Jun N-terminal kinase (JNK), and phosphorylated p38. In IBD, colonic macrophages predominantly exhibit the proinflammatory M1 phenotype, which drives disease progression through the secretion of mediators such as IL-1β, IL-6, IL-12α, IL-23, and TNF (Zhao et al., 2022). Classically activated macrophage (M1 macrophages) are polarized by Th1 cytokines (e.g., interferon-γ) and Toll-like receptor (TLR) ligands (e.g., LPS). In DSS-induced colitis, ERK signaling is activated, which is correlated with increased M1 macrophage infiltration (**Figure 4**).

LPS binds to TLR4, triggering MAPK pathway activation and upregulating proinflammatory mediators, including TNF-α, NO, inducible NO synthase (iNOS), and cyclooxygenase-2 (COX-2) (**Figure 4**). FCD dose-dependently suppressed ERK1/2 and p38 phosphorylation, reduced iNOS and COX-2 protein levels, and attenuated TNF-α and IL-1β production, demonstrating potent MAPK pathway inhibition.

FCD in the MAPK pathway remains relatively unexplored, particularly in UC. However, FCD’s anti-inflammatory effects via MAPK inhibition are established. Junhan Cao et al. investigated sea cucumber-derived FCD, demonstrating its suppression of Helicobacter pylori-induced gastritis through MAPK/NF-κB signaling and gut microbiota modulation. Specifically, FCD significantly enhanced biosynthesis of microbial metabolites including butyrate, isobutyrate, hexanoate, and phospholipids, thereby attenuating gastric inflammation (Cao et al., 2025).

### 5.3 Toll-like receptor pathway

TLRs constitute an essential class of PRRs that orchestrate innate immune responses against microbial pathogens (Yang et al., 2015). TLR4, the principal receptor for LPS signaling, regulates cytokine cascades and caspase activation while pathogen-associated molecular patterns (PAMPs) are detected, thereby bridging pathogen detection and inflammatory signaling in innate immunity (Kawasaki and Kawai, 2014). Upon ligand binding, TLR activation initiates intracellular signaling via recruitment of the adaptor protein myeloid differentiation primary response 88 (MyD88), enabling IRAK-4 association with the receptor–ligand complex. This cascade activates the IκB kinase (IKK) complex, triggering IκB phosphorylation and proteasomal degradation, which enables NF-κB nuclear translocation and subsequent transcription of proinflammatory genes (**Figure 4**) (Kawasaki and Kawai, 2014; Vaamonde-García et al., 2021). FCD derived from Ascophyllum nodosum suppresses LPS-induced macrophage inflammation by inhibiting TLR4/NF-κB signaling (**Figure 4**). Specifically, FCD attenuated LPS-stimulated NO production (IC50: 27.82 μg/mL), iNOS, and COX-2 expression in RAW 264.7 macrophages in a dose-dependent manner (Fernando et al., 2017).

### 5.4 FCD inhibits lipid peroxidation damage

ROS, including superoxide, hydroxyl radicals, hydroperoxyl radicals, NO, and singlet oxygen, are key mediators of oxidative damage in inflamed tissues (Bassoy et al., 2021). ROS amplify inflammation by upregulating factors linked to innate and adaptive immune responses, exacerbating mucosal injury (Tian et al., 2017). Sustained ROS release in inflammatory microenvironments induces cytosolic and metabolic dysfunction, driving tissue damage through lipid peroxidation (LPx), enzyme inactivation, and DNA oxidation (Schieber and Chandel, 2014; Esmaeeli et al., 2023; Liu et al., 2023). In IBD, oxidative stress (OS) arises from an imbalance between oxidant production and antioxidant defenses (Bourgonje et al., 2020). ROS directly activate proinflammatory signaling pathways, including the NF-κB, MAPK, and signal transducer and activator of transcription 3 (STAT3) pathways. Leukocyte infiltration in inflamed mucosa further amplifies ROS generation, perpetuating cellular and tissue injury (Wang et al., 2016).

Excessive ROS production induces compensatory antioxidant responses to counteract oxidative damage. The NADPH oxidase (NOX) family serves as the primary enzymatic generator of ROS in mammalian cells, with NF-κB activation driven by ROS-dependent activation of the IκB kinase α/β (IKKα/β) complex (**Figure 4**). Cyclophosphamide (CPA), an immunosuppressant employed in cancer therapy, paradoxically exacerbates oxidative stress through ROS overproduction and the downregulation of antioxidant enzymes—including catalase (CAT), superoxide dismutase (SOD), and glutathione peroxidase (GSH-Px)—in the spleen and thyroid tissues (Xu and Zhang, 2015; Li et al., 2017). ROS disrupt intestinal epithelial barrier integrity by redistributing TJs and AJs proteins through PKC-, MAPK-, JNK-, and ERK-dependent mechanisms (**Figure 4**). FCD counteracts these effects by inhibiting ROS-driven lipid peroxidation. For example, polysaccharide fractions from the seaweed Solieria filiformis exhibit dose-dependent antioxidant activity, scavenging 2,2-diphenyl-1-picrylhydrazyl (DPPH) free radicals with an IC50 of 1.77 mg/mL (Sousa et al., 2016).

### 5.5 Role of FCD in UC

FCD ameliorates intestinal disorders by restoring intestinal barrier integrity, attenuating lipid peroxidation, and suppressing proinflammatory mediators. FCD modulates the gut microbiota composition, enhances SCFAs production by commensal bacteria, and reverses dysbiosis in UC patients, thereby mitigating disease progression. Through synergistic interactions with the gut microbiota, FCD inhibits the NF-κB and MAPK signaling pathways, downregulates proinflammatory cytokines (e.g., IL-12, IL-23, IL-6, IL-36γ, and IL-13), and counteracts oxidative stress-induced intestinal damage by reducing ROS accumulation. Concurrently, FCD upregulates TJs proteins, MUC2, and sIgA, collectively enhancing mucosal protection in UC.

## 6 Conclusion

This review synthesizes current knowledge on IBD etiology, UC pathogenesis, and the therapeutic mechanisms of FCD in modulating the gut microbiota and inflammation. Dysregulated host–microbiota interactions, particularly in UC, drive immune dysfunction and sustain the upregulation of proinflammatory factors, exacerbating disease severity. FCD acts as a prebiotic agent, restoring intestinal epithelial homeostasis, suppressing inflammation, neutralizing oxidative stress, and rectifying microbial dysbiosis, all of which contribute to its beneficial effects on UC. Despite these advances, key challenges remain: the precise etiology of IBD is unresolved, the structural heterogeneity of FCD (which is dependent on algal species and extraction methods) complicates standardization, and scalable production of low-molecular-weight FCD (LMWF) and identification of specific prebiotic targets for IBD require further investigation. Owing to its anti-inflammatory, antitumor, and immunomodulatory properties, FCD holds promise as a functional food or therapeutic agent for managing IBD and associated malignancies.

The limited clinical translation of FCD as a therapeutic agent stems primarily from its poor bioavailability. Historically, this sulfated polysaccharide has been utilized in traditional Chinese medicine (TCM) to treat diverse pathologies (Shen et al., 2018). In oncology, preclinical studies have extensively investigated FCD’s anticancer properties, with robust evidence demonstrating its dose-dependent cytotoxic effects on colon cancer cells (HT29, Caco-2) via ROS-mediated apoptosis induction (Vishchuk et al., 2013; Usoltseva et al., 2017; Narayani et al., 2019). LMWF has been used as a complementary therapy for patients with metastatic colorectal cancer (Tsai et al., 2017). Despite its suboptimal bioavailability, significant efforts have focused on structural modifications—including cross-linking, ionic interactions, covalent bonding, electrostatic stabilization, and physical entrapment—to enhance its bioactivity. Encapsulation strategies, particularly nanoparticle (NP)-based drug delivery systems, improve FCD’s stability and targeted release at lesion sites (Deepika et al., 2019). Nevertheless, critical gaps persist in pharmacokinetic profiling and bioavailability optimization, requiring further experimental validation to advance clinical translation. Notably, FCD exhibits extraordinary chemopreventive potential due to its pleiotropic modulation of both classical and non-classical signaling pathways.

Currently FDA-approved therapeutics for UC primarily comprise traditional aminosalicylates and glucocorticoids, yet their associated adverse effects and complications remain inadequately resolved (D’Haens, 2016). Consequently, identifying and developing low-toxicity therapeutics with reduced side-effect profiles represents a critical research priority. Polyphenols and polysaccharides, natural products exhibiting anti-inflammatory and antioxidant properties with favorable toxicity profiles at appropriate doses, are considered promising candidates for inflammatory disorders. However, limitations in bioavailability and undefined safety dosage ranges restrict their clinical translation.

Nanoparticle-based drug delivery has emerged as a prominent strategy to address these challenges. Utilizing targeted carriers enables selective drug release at disease sites, minimizes systemic absorption, prevents off-target delivery to healthy tissues, enhances bioavailability, and reduces adverse effects. Mingxiao Cui et al. demonstrated enhanced therapeutic efficacy in UC through nano-encapsulated delivery of Phragmites polysaccharide (RP) to the intestinal tract (Cui et al., 2022). In a comprehensive review, Cui et al. summarize polysaccharide delivery via nanocarriers, highlighting that encapsulation protects polysaccharides from gastrointestinal degradation, improves stability, promotes localization to inflamed intestinal regions, reduces side effects, and enhances bioavailability through targeted colonic delivery (Cui et al., 2021).

While nanocarrier systems represent one approach to improve polysaccharide bioavailability, alternative strategies—including chemical modification and enzyme inhibitors—constitute emerging UC therapeutics. Further clinical validation is required to assess their applicability.

## References

[B1] Ahmad T. Eapen M. S. Ishaq M. Park A. Y. Karpiniec S. S. Stringer D. N.. (2021). Anti-inflammatory activity of fucoidan extracts *in vitro* . Mar. Drugs 19 (12), 702. doi: 10.3390/md19120702, PMID: 34940701 PMC8704339

[B2] Akhtar M. Chen Y. Ma Z. Zhang X. Shi D. Khan J. A.. (2022). Gut microbiota-derived short chain fatty acids are potential mediators in gut inflammation. Anim. Nutr. 8, 350–360. doi: 10.1016/j.aninu.2021.11.005, PMID: 35510031 PMC9040132

[B3] Ale M. T. Mikkelsen J. D. Meyer A. S. (2011). Important determinants for fucoidan bioactivity: a critical review of structure-function relations and extraction methods for fucose-containing sulfated polysaccharides from brown seaweeds. Mar. Drugs 9, 2106–2130. doi: 10.3390/md9102106, PMID: 22073012 PMC3210621

[B4] Apostolova E. Lukova P. Baldzhieva A. Katsarov P. Nikolova M. Iliev I.. (2020). Immunomodulatory and anti-inflammatory effects of fucoidan: A review. Polymers (Basel) 12 (10), 2338. doi: 10.3390/polym12102338, PMID: 33066186 PMC7602053

[B5] Asanka Sanjeewa K. K. Jayawardena T. U. Kim H. S. Kim S. Y. Shanura Fernando I. P. Wang L.. (2019). Fucoidan isolated from Padina commersonii inhibit LPS-induced inflammation in macrophages blocking TLR/NF-κB signal pathway. Carbohydr Polym. 224, 115195. doi: 10.1016/j.carbpol.2019.115195, PMID: 31472848

[B6] Alatab S Sepanlou SG Ikuta K Vahedi H Bisignano C Safiri S. (2020). The global, regional, and national burden of inflammatory bowel disease in 195 countries and territories, 1990-2017: a systematic analysis for the Global Burden of Disease Study 2017. Lancet Gastroenterol. Hepatol. 5, 17–30. doi: 10.1016/S2468-1253(19)30333-4, PMID: 31648971 PMC7026709

[B7] Bassoy E. Y. Walch M. Martinvalet D. (2021). Reactive oxygen species: do they play a role in adaptive immunity? Front. Immunol. 12, 755856. doi: 10.3389/fimmu.2021.755856, PMID: 34899706 PMC8653250

[B8] Becker H. E. F. Demers K. Derijks L. J. J. Jonkers D. M. A. E. Penders J. (2023). Current evidence and clinical relevance of drug-microbiota interactions in inflammatory bowel disease. Front. Microbiol. 14. doi: 10.3389/fmicb.2023.1107976, PMID: 36910207 PMC9996055

[B9] Bhat A. A. Uppada S. Achkar I. W. Hashem S. Yadav S. K. Shanmugakonar M.. (2018). Tight junction proteins and signaling pathways in cancer and inflammation: A functional crosstalk. Front. Physiol. 9, 1942. doi: 10.3389/fphys.2018.01942, PMID: 30728783 PMC6351700

[B10] Bishop J. L. Roberts M. E. Beer J. L. Huang M. Chehal M. K. Fan X.. (2014). Lyn activity protects mice from DSS colitis and regulates the production of IL-22 from innate lymphoid cells. Mucosal Immunol. 7, 405–416. doi: 10.1038/mi.2013.60, PMID: 24045577

[B11] Böhm L. Torsin S. Tint S. H. Eckstein M. T. Ludwig T. Pérez J. C. (2017). The yeast form of the fungus Candida albicans promotes persistence in the gut of gnotobiotic mice. PloS Pathog. 13, e1006699. doi: 10.1371/journal.ppat.1006699, PMID: 29069103 PMC5673237

[B12] Bourgonje A. R. Feelisch M. Faber K. N. Pasch A. Dijkstra G. van Goor H. (2020). Oxidative stress and redox-modulating therapeutics in inflammatory bowel disease. Trends Mol. Med. 26, 1034–1046. doi: 10.1016/j.molmed.2020.06.006, PMID: 32620502

[B13] Braicu C. Buse M. Busuioc C. Drula R. Gulei D. Raduly L.. (2019). A comprehensive review on MAPK: A promising therapeutic target in cancer. Cancers (Basel) 11 (10), 1618. doi: 10.3390/cancers11101618, PMID: 31652660 PMC6827047

[B14] Cao J. Yao M. Wang K. Qin L. Zhang Q. Zhang H.. (2025). Sea Cucumber Fucoidan Inhibits Helicobacter pylori Gastritis via MAPK/NF-κB Signaling and Gut Microbiota Modulation. J. Agric. Food Chem. 73 (23), 14333–14352. doi: 10.1021/acs.jafc.5c02190, PMID: 40444701

[B15] Chen A. Liu Y. Zhang T. Xiao Y. Xu X. Xu Z.. (2023). Chain conformation, mucoadhesive properties of fucoidan in the gastrointestinal tract and its effects on the gut microbiota. Carbohydr. Polymers. 304, 120460. doi: 10.1016/j.carbpol.2022.120460, PMID: 36641186

[B16] Cho Y. H. Renouf M. J. Omotoso O. McPhee J. B. (2022). Inflammatory bowel disease-associated adherent-invasive Escherichia coli have elevated host-defense peptide resistance. FEMS Microbiol. Lett. 369 (1), fnac098. doi: 10.1093/femsle/fnac098, PMID: 36208952

[B17] Corino C. Di Giancamillo A. Modina S. C. Rossi R. (2021). Prebiotic effects of seaweed polysaccharides in pigs. Anim. (Basel). 11 (6), 1573. doi: 10.3390/ani11061573, PMID: 34072221 PMC8229765

[B18] Cui M. Fang Z. Song M. Zhou T. Wang Y. Liu K. (2022). Phragmites rhizoma polysaccharide-based nanocarriers for synergistic treatment of ulcerative colitis. Int. J. Biol. Macromol. 220, 22–32. doi: 10.1016/j.ijbiomac.2022.07.245, PMID: 35932810

[B19] Cui D. Ma J. Liang T. Sun L. Meng L. Liang T.. (2019). Selenium nanoparticles fabricated in laminarin polysaccharides solutions exert their cytotoxicities in HepG2 cells by inhibiting autophagy and promoting apoptosis. Int. J. Biol. Macromol. 137, 829–835. doi: 10.1016/j.ijbiomac.2019.07.031, PMID: 31284007

[B20] Cui M. Zhang M. Liu K. (2021). Colon-targeted drug delivery of polysaccharide-based nanocarriers for synergistic treatment of inflammatory bowel disease: A review. Carbohydr. Polymers. 272, 118530. doi: 10.1016/j.carbpol.2021.118530, PMID: 34420762

[B21] D’Haens G. (2016). Systematic review: second-generation vs. conventional corticosteroids for induction of remission in ulcerative colitis. Aliment Pharmacol. Ther. 44, 1018–1029. doi: 10.1111/apt.13803, PMID: 27650488

[B22] Deepika M. S. Thangam R. Sheena T. S. Sasirekha R. Sivasubramanian S. Babu M. D.. (2019). A novel rutin-fucoidan complex based phytotherapy for cervical cancer through achieving enhanced bioavailability and cancer cell apoptosis. Biomedicine Pharmacotherapy. 109, 1181–1195. doi: 10.1016/j.biopha.2018.10.178, PMID: 30551368

[B23] De Marco S. Sichetti M. Muradyan D. Piccioni M. Traina G. Pagiotti R.. (2018). Probiotic cell-free supernatants exhibited anti-inflammatory and antioxidant activity on human gut epithelial cells and macrophages stimulated with LPS. Evid Based Complement Alternat Med. 2018, 1756308. doi: 10.1155/2018/1756308, PMID: 30069221 PMC6057331

[B24] Devkota S. Wang Y. Musch M. W. Leone V. Fehlner-Peach H. Nadimpalli A.. (2012). Dietary-fat-induced taurocholic acid promotes pathobiont expansion and colitis in Il10-/- mice. Nature. 487, 104–108. doi: 10.1038/nature11225, PMID: 22722865 PMC3393783

[B25] Dewayani A. Fauzia K. A. Alfaray R. I. Waskito L. A. Doohan D. Rezkitha Y. A. A.. (2021). The roles of IL-17, IL-21, and IL-23 in the helicobacter pylori infection and gastrointestinal inflammation: A review. Toxins. 13, 315. doi: 10.3390/toxins13050315, PMID: 33924897 PMC8147029

[B26] Doron I. Leonardi I. Li X. V. Fiers W. D. Semon A. Bialt-DeCelie M.. (2021). Human gut mycobiota tune immunity via CARD9-dependent induction of anti-fungal IgG antibodies. Cell. 184, 1017–31.e14. doi: 10.1016/j.cell.2021.01.016, PMID: 33548172 PMC7936855

[B27] Esmaeeli M. Nimtz M. Jänsch L. Ruddock L. W. Leimkühler S. (2023). Mechanistic insights into the ROS-mediated inactivation of human aldehyde oxidase. FEBS Lett. 597, 1792–1801. doi: 10.1002/1873-3468.14669, PMID: 37247262

[B28] Fan LJ X. Y. Wang Y. X. Han D. D. Liu Y. L. Li J. H. Fu J.. (2023). Gut microbiota bridges dietary nutrients and host immunity. Sci. China-Life Sci. 66, 2466–2514. doi: 10.1007/s11427-023-2346-1, PMID: 37286860 PMC10247344

[B29] Fawad J. A. Luzader D. H. Hanson G. F. Moutinho T. J. Jr. McKinney C. A. Mitchell P. G.. (2022). Histone deacetylase inhibition by gut microbe-generated short-chain fatty acids entrains intestinal epithelial circadian rhythms. Gastroenterology. 163, 1377–90.e11. doi: 10.1053/j.gastro.2022.07.051, PMID: 35934064 PMC11551968

[B30] Fernando I. P. S. Sanjeewa K. K. A. Samarakoon K. W. Lee W. W. Kim H. S. Kang N.. (2017). A fucoidan fraction purified from Chnoospora minima; a potential inhibitor of LPS-induced inflammatory responses. Int. J. Biol. Macromol 104, 1185–1193. doi: 10.1016/j.ijbiomac.2017.07.031, PMID: 28690171

[B31] Forbes J. D. Chen C.-Y. Knox N. C. Marrie R.-A. El-Gabalawy H. de Kievit T.. (2018). A comparative study of the gut microbiota in immune-mediated inflammatory diseases—does a common dysbiosis exist? Microbiome 6, 221. doi: 10.1186/s40168-018-0603-4, PMID: 30545401 PMC6292067

[B32] Gevers D. Kugathasan S. Denson L. A. Vázquez-Baeza Y. Van Treuren W. Ren B.. (2014). The treatment-naive microbiome in new-onset Crohn’s disease. Cell Host Microbe 15, 382–392. doi: 10.1016/j.chom.2014.02.005, PMID: 24629344 PMC4059512

[B33] Guo C. Wang Y. Zhang S. Zhang X. Du Z. Li M.. (2021). Crataegus pinnatifida polysaccharide alleviates colitis via modulation of gut microbiota and SCFAs metabolism. Int. J. Biol. Macromol. 181, 357–368. doi: 10.1016/j.ijbiomac.2021.03.137, PMID: 33774071

[B34] Hidalgo-Vico S. Prieto D. Alonso-Monge R. Román E. Maufrais C. d’Enfert C.. (2024). Candida albicans strains adapted to the mouse gut are resistant to bile salts via a Flo8-dependent mechanism. Fungal Genet. Biol. 175, 103939. doi: 10.1016/j.fgb.2024.103939, PMID: 39486612

[B35] Hu Z. Yang M. Ye Q. Qin K. Wu M. Gu R.. (2018). Tou nong san attenuates inflammation in TNBS-IBD model by inhibiting NF-κB signaling pathway. Evid Based Complement Alternat Med. 2018, 6929307. doi: 10.1155/2018/6929307, PMID: 30046345 PMC6036830

[B36] Ikeda-Ohtsubo W. López Nadal A. Zaccaria E. Iha M. Kitazawa H. Kleerebezem M.. (2020). Intestinal microbiota and immune modulation in zebrafish by fucoidan from okinawa mozuku (Cladosiphon okamuranus). Front. Nutr. 7, 67. doi: 10.3389/fnut.2020.00067, PMID: 32671088 PMC7327095

[B37] Iraha A. Chinen H. Hokama A. Yonashiro T. Kinjo T. Kishimoto K.. (2013). Fucoidan enhances intestinal barrier function by upregulating the expression of claudin-1. World J. Gastroenterol. 19, 5500–5507. doi: 10.3748/wjg.v19.i33.5500, PMID: 24023493 PMC3761103

[B38] Jayawardena T. U. Nagahawatta D. P. Fernando I. P. S. Kim Y. T. Kim J. S. Kim W. S.. (2022). A review on fucoidan structure, extraction techniques, and its role as an immunomodulatory agent. Mar. Drugs 20 (12), 755. doi: 10.3390/md20120755, PMID: 36547902 PMC9782291

[B39] Jia H. Li Y. Zheng Y. Wang H. Zhao F. Yang X.. (2025). Recent advances in fucoidan-based improved delivery systems: Structure, carrier types and biomedical applications. Carbohydr Polym. 352, 123183. doi: 10.1016/j.carbpol.2024.123183, PMID: 39843086

[B40] Jia D. J. Wang Q. W. Hu Y. Y. He J. M. Ge Q. W. Qi Y. D.. (2022). Lactobacillus johnsonii alleviates colitis by TLR1/2-STAT3 mediated CD206(+) macrophages(IL-10) activation. Gut Microbes 14, 2145843. doi: 10.1080/19490976.2022.2145843, PMID: 36398889 PMC9677986

[B41] Jiang MZ L. C. Xu C. Jiang H. Wang Y. L. Liu S. J. (2024). Gut microbial interactions based on network construction and bacterial pairwise cultivation. Sci. China-Life Sci. 67 (8), 1751–1762. doi: 10.1007/s11427-023-2537-0, PMID: 38600293

[B42] Jin W. Wu W. Tang H. Wei B. Wang H. Sun J.. (2019). Structure analysis and anti-tumor and anti-angiogenic activities of sulfated galactofucan extracted from sargassum thunbergii. Mar. Drugs 17 (1), 52. doi: 10.3390/md17010052, PMID: 30641954 PMC6356460

[B43] Kadena K. Tomori M. Iha M. Nagamine T. (2018). Absorption study of mozuku fucoidan in Japanese volunteers. Mar. Drugs 16 (8), 254. doi: 10.3390/md16080254, PMID: 30061499 PMC6117716

[B44] Kawai T. Akira S. (2011). Toll-like receptors and their crosstalk with other innate receptors in infection and immunity. Immunity. 34, 637–650. doi: 10.1016/j.immuni.2011.05.006, PMID: 21616434

[B45] Kawasaki T. Kawai T. (2014). Toll-like receptor signaling pathways. Front. Immunol. 5, 461. doi: 10.3389/fimmu.2014.00461, PMID: 25309543 PMC4174766

[B46] Koh H. S. A. Lu J. Zhou W. (2019). Structure characterization and antioxidant activity of fucoidan isolated from Undaria pinnatifida grown in New Zealand. Carbohydr Polym. 212, 178–185. doi: 10.1016/j.carbpol.2019.02.040, PMID: 30832845

[B47] Kong Y. Olejar K. J. On S. L. W. Chelikani V. (2020). The potential of lactobacillus spp. for modulating oxidative stress in the gastrointestinal tract. Antioxidants (Basel). 9 (7), 610. doi: 10.3390/antiox9070610, PMID: 32664392 PMC7402165

[B48] Lajili S. Ammar H. H. Mzoughi Z. Amor H. B. H. Muller C. D. Majdoub H.. (2019). Characterization of sulfated polysaccharide from Laurencia obtusa and its apoptotic, gastroprotective and antioxidant activities. Int. J. Biol. Macromol. 126, 326–336. doi: 10.1016/j.ijbiomac.2018.12.089, PMID: 30543883

[B49] Lakshmana Senthil S. (2024). A comprehensive review to assess the potential, health benefits and complications of fucoidan for developing as functional ingredient and nutraceutical. Int. J. Biol. Macromol 277, 134226. doi: 10.1016/j.ijbiomac.2024.134226, PMID: 39074709

[B50] Lean Q. Y. Eri R. D. Fitton J. H. Patel R. P. Gueven N. (2015). Fucoidan extracts ameliorate acute colitis. PloS One 10, e0128453. doi: 10.1371/journal.pone.0128453, PMID: 26083103 PMC4471193

[B51] Le Berre C. Honap S. Peyrin-Biroulet L. (2023). Ulcerative colitis. Lancet. 402, 571–584. doi: 10.1016/S0140-6736(23)00966-2, PMID: 37573077

[B52] Lee Y. S. Kim T. Y. Kim Y. Lee S. H. Kim S. Kang S. W.. (2018). Microbiota-derived lactate accelerates intestinal stem-cell-mediated epithelial development. Cell Host Microbe 24, 833–46.e6. doi: 10.1016/j.chom.2018.11.002, PMID: 30543778

[B53] Li X. V. Leonardi I. Putzel G. G. Semon A. Fiers W. D. Kusakabe T.. (2022). Immune regulation by fungal strain diversity in inflammatory bowel disease. Nature. 603, 672–678. doi: 10.1038/s41586-022-04502-w, PMID: 35296857 PMC9166917

[B54] Li W. J. Li L. Zhen W. Y. Wang L. F. Pan M. Lv J. Q.. (2017). Ganoderma atrum polysaccharide ameliorates ROS generation and apoptosis in spleen and thymus of immunosuppressed mice. Food Chem. Toxicol. 99, 199–208. doi: 10.1016/j.fct.2016.11.033, PMID: 27913287

[B55] Li R. Mou J. Zhao L. Hu M. Wang B. Sun Y.. (2024). Fucoidan from Stichopus chloronotus relieved DSS induced ulcerative colitis through inhibiting intestinal barrier disruption and oxidative stress. Int. J. Biol. Macromolecules. 283, 137811. doi: 10.1016/j.ijbiomac.2024.137811, PMID: 39566803

[B56] Li S. Qian Q. Yang H. Wu Z. Xie Y. Yin Y.. (2024). Fucoidan alleviated dextran sulfate sodium–induced ulcerative colitis with improved intestinal barrier, reshaped gut microbiota composition, and promoted autophagy in male C57BL/6 mice. Nutr. Res. 122, 1–18. doi: 10.1016/j.nutres.2023.11.009, PMID: 38064857

[B57] Liu J. Han X. Zhang T. Tian K. Li Z. Luo F. (2023). Reactive oxygen species (ROS) scavenging biomaterials for anti-inflammatory diseases: from mechanism to therapy. J. Hematol. Oncol. 16, 116. doi: 10.1186/s13045-023-01512-7, PMID: 38037103 PMC10687997

[B58] Liu J. Tan Y. Cheng H. Zhang D. Feng W. Peng C. (2022). Functions of gut microbiota metabolites, current status and future perspectives. Aging Dis. 13, 1106–1126. doi: 10.14336/AD.2022.0104, PMID: 35855347 PMC9286904

[B59] Liyanage N. M. Lee H. G. Nagahawatta D. P. Jayawardhana H. Song K. M. Choi Y. S.. (2023). Fucoidan from Sargassum autumnale Inhibits Potential Inflammatory Responses via NF-κB and MAPK Pathway Suppression in Lipopolysaccharide-Induced RAW 264.7 Macrophages. Mar. Drugs 21 (7), 374. doi: 10.3390/md21070374, PMID: 37504905 PMC10381575

[B60] Luo J. Wang Z. Fan B. Wang L. Liu M. An Z.. (2021). A comparative study of the effects of different fucoidans on cefoperazone-induced gut microbiota disturbance and intestinal inflammation. Food Funct. 12, 9087–9097. doi: 10.1039/D1FO00782C, PMID: 34388231

[B61] Luthuli S. Wu S. Cheng Y. Zheng X. Wu M. Tong H. (2019). Therapeutic effects of fucoidan: A review on recent studies. Mar. Drugs 17 (9), 487. doi: 10.3390/md17090487, PMID: 31438588 PMC6780838

[B62] Machiels K. Joossens M. Sabino J. De Preter V. Arijs I. Eeckhaut V.. (2014). A decrease of the butyrate-producing species Roseburia hominis and Faecalibacterium prausnitzii defines dysbiosis in patients with ulcerative colitis. Gut. 63, 1275–1283. doi: 10.1136/gutjnl-2013-304833, PMID: 24021287

[B63] Matson V. Chervin C. S. Gajewski T. F. (2021). Cancer and the microbiome-influence of the commensal microbiota on cancer, immune responses, and immunotherapy. Gastroenterology. 160, 600–613. doi: 10.1053/j.gastro.2020.11.041, PMID: 33253684 PMC8409239

[B64] Matsumoto S. Nagaoka M. Hara T. Kimura-Takagi I. Mistuyama K. Ueyama S. (2004). Fucoidan derived from Cladosiphon okamuranus Tokida ameliorates murine chronic colitis through the down-regulation of interleukin-6 production on colonic epithelial cells. Clin. Exp. Immunol. 136, 432–439. doi: 10.1111/j.1365-2249.2004.02462.x, PMID: 15147344 PMC1809061

[B65] Ma Y F. Y. Ding S. Jiang H. Fang J. Liu G. (2023). Trace metal elements: a bridge between host and intestinal microorganisms. Sci. China Life Sci. Epub 66, 1976–1993. doi: 10.1007/s11427-022-2359-4, PMID: 37528296

[B66] Ministro P. Martins D. (2017). Fecal biomarkers in inflammatory bowel disease: how, when and why? Expert Rev. Gastroenterol. Hepatol. 11, 317–328. doi: 10.1080/17474124.2017.1292128, PMID: 28276813

[B67] Mirsepasi-Lauridsen H. C. Du Z. Struve C. Charbon G. Karczewski J. Krogfelt K. A.. (2016). Secretion of alpha-hemolysin by escherichia coli disrupts tight junctions in ulcerative colitis patients. Clin. Transl. Gastroenterol. 7 (3), e149. doi: 10.1038/ctg.2016.3, PMID: 26938480 PMC4822097

[B68] Mirsepasi-Lauridsen H. C. Struve C. Petersen A. M. Krogfelt K. A. (2020). Effect of α-hemolysin producing E. coli in two different mouse strains in a DSS model of inflammatory bowel disease. Microorganisms. 8 (12), 1971. doi: 10.3390/microorganisms8121971, PMID: 33322398 PMC7764192

[B69] Mirsepasi-Lauridsen H. C. Vallance B. A. Krogfelt K. A. Petersen A. M. (2019). Escherichia coli pathobionts associated with inflammatory bowel disease. Clin. Microbiol. Rev. 32 (2), e00060-18. doi: 10.1128/CMR.00060-18, PMID: 30700431 PMC6431131

[B70] Moyes D. L. Wilson D. Richardson J. P. Mogavero S. Tang S. X. Wernecke J.. (2016). Candidalysin is a fungal peptide toxin critical for mucosal infection. Nature. 532, 64–68. doi: 10.1038/nature17625, PMID: 27027296 PMC4851236

[B71] Nagamine T. Nakazato K. Tomioka S. Iha M. Nakajima K. (2014). Intestinal absorption of fucoidan extracted from the brown seaweed, Cladosiphon okamuranus. Mar. Drugs 13, 48–64. doi: 10.3390/md13010048, PMID: 25546518 PMC4306924

[B72] Narayani S. S. Saravanan S. Ravindran J. Ramasamy M. S. Chitra J. (2019). *In vitro* anticancer activity of fucoidan extracted from Sargassum cinereum against Caco-2 cells. Int. J. Biol. Macromolecules. 138, 618–628. doi: 10.1016/j.ijbiomac.2019.07.127, PMID: 31344415

[B73] Naser S. A. Sagramsingh S. R. Naser A. S. Thanigachalam S. (2014). Mycobacterium avium subspecies paratuberculosis causes Crohn’s disease in some inflammatory bowel disease patients. World J. Gastroenterol. 20, 7403–7415. doi: 10.3748/wjg.v20.i23.7403, PMID: 24966610 PMC4064085

[B74] Nishino K. Nishida A. Inoue R. Kawada Y. Ohno M. Sakai S.. (2018). Analysis of endoscopic brush samples identified mucosa-associated dysbiosis in inflammatory bowel disease. J. Gastroenterol. 53, 95–106. doi: 10.1007/s00535-017-1384-4, PMID: 28852861

[B75] Nya E. (2022). “Factors influencing the efficacy of probiotics,” in Probiotics in aquaculture (Springer), 263–283. doi: 10.1007/978-3-030-98621-6_13

[B76] Obluchinskaya E. D. Pozharitskaya O. N. Gorshenina E. V. Zakharov D. V. Flisyuk E. V. Terninko I. I.. (2023). Arctic edible brown alga fucus distichus L.: biochemical composition, antiradical potential and human health risk. Plants (Basel) 12 (12), 2380. doi: 10.3390/plants12122380, PMID: 37376005 PMC10301332

[B77] Paone P. Cani P. D. (2020). Mucus barrier, mucins and gut microbiota: the expected slimy partners? Gut 69, 2232–2243. doi: 10.1136/gutjnl-2020-322260, PMID: 32917747 PMC7677487

[B78] Park Y. N. Conway K. Pujol C. Daniels K. J. Soll D. R. (2020). EFG1 Mutations, Phenotypic Switching, and Colonization by Clinical a/α Strains of Candida albicans. mSphere. 5 (1), e00795-19. doi: 10.1128/mSphere.00795-19, PMID: 32024711 PMC7002308

[B79] Peng Z. Wang D. He Y. Wei Z. Xie M. Xiong T. (2024). Gut distribution, impact factor, and action mechanism of bacteriocin-producing beneficial microbes as promising antimicrobial agents in gastrointestinal infection. Probiotics Antimicrob. Proteins. 16, 1516–1527. doi: 10.1007/s12602-024-10222-6, PMID: 38319538

[B80] Pomin V. H. (2015). Sulfated glycans in inflammation. Eur. J. Med. Chem. 92, 353–369. doi: 10.1016/j.ejmech.2015.01.002, PMID: 25576741

[B81] Pozharitskaya O. N. Shikov A. N. Faustova N. M. Obluchinskaya E. D. Kosman V. M. Vuorela H.. (2018). Pharmacokinetic and Tissue Distribution of Fucoidan from Fucus vesiculosus after Oral Administration to Rats. Mar. Drugs 16 (4), 132. doi: 10.3390/md16040132, PMID: 29669995 PMC5923419

[B82] Reenaers C. Bossuyt P. Hindryckx P. Vanpoucke H. Cremer A. Baert F. (2018). Expert opinion for use of faecal calprotectin in diagnosis and monitoring of inflammatory bowel disease in daily clinical practice. United Eur. Gastroenterol. J. 6, 1117–1125. doi: 10.1177/2050640618784046, PMID: 30288273 PMC6169045

[B83] Sanjeewa K. K. A. Lee J. S. Kim W. S. Jeon Y. J. (2017). The potential of brown-algae polysaccharides for the development of anticancer agents: An update on anticancer effects reported for fucoidan and laminaran. Carbohydr Polym. 177, 451–459. doi: 10.1016/j.carbpol.2017.09.005, PMID: 28962791

[B84] Schieber M. Chandel N. S. (2014). ROS function in redox signaling and oxidative stress. Curr. Biol. 24, R453–R462. doi: 10.1016/j.cub.2014.03.034, PMID: 24845678 PMC4055301

[B85] Schoeler M. Caesar R. (2019). Dietary lipids, gut microbiota and lipid metabolism. Rev. Endocr. Metab. Disord. 20, 461–472. doi: 10.1007/s11154-019-09512-0, PMID: 31707624 PMC6938793

[B86] Shang Q. Shan X. Cai C. Hao J. Li G. Yu G. (2016). Dietary fucoidan modulates the gut microbiota in mice by increasing the abundance of Lactobacillus and Ruminococcaceae. Food Funct. 7, 3224–3232. doi: 10.1039/C6FO00309E, PMID: 27334000

[B87] Shao T. Y. Haslam D. B. Bennett R. J. Way S. S. (2022a). Friendly fungi: symbiosis with commensal Candida albicans. Trends Immunol. 43, 706–717. doi: 10.1016/j.it.2022.07.003, PMID: 35961916 PMC10027380

[B88] Shao T. Y. Kakade P. Witchley J. N. Frazer C. Murray K. L. Ene I. V.. (2022b). Candida albicans oscillating UME6 expression during intestinal colonization primes systemic Th17 protective immunity. Cell Rep. 39, 110837. doi: 10.1016/j.celrep.2022.110837, PMID: 35584674 PMC9196946

[B89] Sharma G. Garg N. Hasan S. Shirodkar S. (2022). Prevotella: An insight into its characteristics and associated virulence factors. Microb. Pathog. 169, 105673. doi: 10.1016/j.micpath.2022.105673, PMID: 35843443

[B90] Shen P. Yin Z. Qu G. Wang C. (2018). “11 - fucoidan and its health benefits,” in Bioactive seaweeds for food applications. Ed. Qin Y. (Academic Press), 223–238. doi: 10.1016/B978-0-12-813312-5.00011-X

[B91] Shi H. Chang Y. Gao Y. Wang X. Chen X. Wang Y.. (2017). Dietary fucoidan of Acaudina molpadioides alters gut microbiota and mitigates intestinal mucosal injury induced by cyclophosphamide. Food Funct. 8, 3383–3393. doi: 10.1039/C7FO00932A, PMID: 28861559

[B92] Sicard J. F. Le Bihan G. Vogeleer P. Jacques M. Harel J. (2017). Interactions of intestinal bacteria with components of the intestinal mucus. Front. Cell Infect. Microbiol. 7, 387. doi: 10.3389/fcimb.2017.00387, PMID: 28929087 PMC5591952

[B93] Sittipo P. Shim J. W. Lee Y. K. (2019). Microbial metabolites determine host health and the status of some diseases. Int. J. Mol. Sci. 20 (21), 5296. doi: 10.3390/ijms20215296, PMID: 31653062 PMC6862038

[B94] Smith A. J. Graves B. Child R. Rice P. J. Ma Z. Lowman D. W.. (2018). Immunoregulatory activity of the natural product laminarin varies widely as a result of its physical properties. J. Immunol. 200, 788–799. doi: 10.4049/jimmunol.1701258, PMID: 29246954 PMC5760317

[B95] Smith P. M. Howitt M. R. Panikov N. Michaud M. Gallini C. A. Bohlooly Y. M.. (2013). The microbial metabolites, short-chain fatty acids, regulate colonic Treg cell homeostasis. Science. 341, 569–573. doi: 10.1126/science.1241165, PMID: 23828891 PMC3807819

[B96] Sokol H. Conway K. L. Zhang M. Choi M. Morin B. Cao Z.. (2013). Card9 mediates intestinal epithelial cell restitution, T-helper 17 responses, and control of bacterial infection in mice. Gastroenterology. 145, 591–601.e3. doi: 10.1053/j.gastro.2013.05.047, PMID: 23732773 PMC3781941

[B97] Sousa W. M. Silva R. O. Bezerra F. F. Bingana R. D. Barros F. C. N. Costa L. E. C.. (2016). Sulfated polysaccharide fraction from marine algae Solieria filiformis: Structural characterization, gastroprotective and antioxidant effects. Carbohydr Polym. 152, 140–148. doi: 10.1016/j.carbpol.2016.06.111, PMID: 27516258

[B98] Sun M. Liu Y. Song Y. Gao Y. Zhao F. Luo Y.. (2020). The ameliorative effect of Lactobacillus plantarum-12 on DSS-induced murine colitis. Food Funct. 11, 5205–5222. doi: 10.1039/D0FO00007H, PMID: 32458908

[B99] Sun M. Wu W. Chen L. Yang W. Huang X. Ma C.. (2018). Microbiota-derived short-chain fatty acids promote Th1 cell IL-10 production to maintain intestinal homeostasis. Nat. Commun. 9, 3555. doi: 10.1038/s41467-018-05901-2, PMID: 30177845 PMC6120873

[B100] Takahashi M. Takahashi K. Abe S. Yamada K. Suzuki M. Masahisa M.. (2020). Improvement of psoriasis by alteration of the gut environment by oral administration of fucoidan from cladosiphon okamuranus. Mar. Drugs 18 (3), 154. doi: 10.3390/md18030154, PMID: 32164223 PMC7143489

[B101] Tang C. Ding R. Sun J. Liu J. Kan J. Jin C. (2019). The impacts of natural polysaccharides on intestinal microbiota and immune responses - a review. Food Funct. 10, 2290–2312. doi: 10.1039/C8FO01946K, PMID: 31032827

[B102] Tian T. Wang Z. Zhang J. (2017). Pathomechanisms of oxidative stress in inflammatory bowel disease and potential antioxidant therapies. Oxid. Med. Cell Longev. 2017, 4535194. doi: 10.1155/2017/4535194, PMID: 28744337 PMC5506473

[B103] Torode T. A. Marcus S. E. Jam M. Tonon T. Blackburn R. S. Hervé C.. (2015). Monoclonal antibodies directed to fucoidan preparations from brown algae. PloS One 10, e0118366. doi: 10.1371/journal.pone.0118366, PMID: 25692870 PMC4333822

[B104] Tsai H. L. Tai C. J. Huang C. W. Chang F. R. Wang J. Y. (2017). Efficacy of low-molecular-weight fucoidan as a supplemental therapy in metastatic colorectal cancer patients: A double-blind randomized controlled trial. Mar. Drugs 15 (4), 122. doi: 10.3390/md15040122, PMID: 28430159 PMC5408268

[B105] Usoltseva R. V. Anastyuk S. D. Shevchenko N. M. Surits V. V. Silchenko A. S. Isakov V. V.. (2017). Polysaccharides from brown algae Sargassum duplicatum: the structure and anticancer activity *in vitro* . Carbohydr. Polymers 175, 547–556. doi: 10.1016/j.carbpol.2017.08.044, PMID: 28917899

[B106] Usoltseva R. V. Anastyuk S. D. Surits V. V. Shevchenko N. M. Thinh P. D. Zadorozhny P. A.. (2019). Comparison of structure and *in vitro* anticancer activity of native and modified fucoidans from Sargassum feldmannii and S. duplicatum. Int. J. Biol. Macromol. 124, 220–228. doi: 10.1016/j.ijbiomac.2018.11.223, PMID: 30496854

[B107] Vaamonde-García C. Flórez-Fernández N. Torres M. D. Lamas-Vázquez M. J. Blanco F. J. Domínguez H.. (2021). Study of fucoidans as natural biomolecules for therapeutical applications in osteoarthritis. Carbohydr Polym. 258, 117692. doi: 10.1016/j.carbpol.2021.117692, PMID: 33593565

[B108] Veiga P. Gallini C. A. Beal C. Michaud M. Delaney M. L. DuBois A.. (2010). Bifidobacterium animalis subsp. lactis fermented milk product reduces inflammation by altering a niche for colitogenic microbes. Proc. Natl. Acad. Sci. U S A. 107, 18132–18137. doi: 10.1073/pnas.1011737107, PMID: 20921388 PMC2964251

[B109] Vishchuk O. S. Ermakova S. P. Zvyagintseva T. N. (2013). The effect of sulfated (1→3)-α-l-fucan from the brown alga Saccharina cichorioides Miyabe on resveratrol-induced apoptosis in colon carcinoma Cells. Mar. Drugs 11, 194–212. doi: 10.3390/md11010194, PMID: 23337253 PMC3564167

[B110] Wakade R. S. Ristow L. C. Wellington M. Krysan D. J. (2023). Intravital imaging-based genetic screen reveals the transcriptional network governing Candida albicans filamentation during mammalian infection. Elife. 12, e85114. doi: 10.7554/eLife.85114.sa2, PMID: 36847358 PMC9995110

[B111] Walczuk U. Sobieszczańska B. Turniak M. Rzeszutko M. Duda-Madej A. Iwańczak B. (2019). The prevalence of mucosa-associated diffusely adherent Escherichia coli in children with inflammatory bowel disease. Adv. Clin. Exp. Med. 28, 899–905. doi: 10.17219/acem/94149, PMID: 31066244

[B112] Wang L. Ai C. Wen C. Qin Y. Liu Z. Wang L.. (2020). Fucoidan isolated from Ascophyllum nodosum alleviates gut microbiota dysbiosis and colonic inflammation in antibiotic-treated mice. Food Funct. 11, 5595–5606. doi: 10.1039/D0FO00668H, PMID: 32525182

[B113] Wang Z. Li S. Cao Y. Tian X. Zeng R. Liao D. F.. (2016). Oxidative stress and carbonyl lesions in ulcerative colitis and associated colorectal cancer. Oxid. Med. Cell Longev. 2016, 9875298. doi: 10.1155/2016/9875298, PMID: 26823956 PMC4707327

[B114] Wang F. Xiao Y. Neupane S. Ptak S. H. Römer R. Xiong J.. (2021). Influence of fucoidan extracts from different fucus species on adult stem cells and molecular mediators in *in vitro* models for bone formation and vascularization. Mar. Drugs 19 (4), 194. doi: 10.3390/md19040194, PMID: 33805470 PMC8066524

[B115] Wang Y. Xing M. Cao Q. Ji A. Liang H. Song S. (2019). Biological activities of fucoidan and the factors mediating its therapeutic effects: A review of recent studies. Mar. Drugs 17 (3), 183. doi: 10.3390/md17030183, PMID: 30897733 PMC6471298

[B116] Wells J. M. Rossi O. Meijerink M. van Baarlen P. (2011). Epithelial crosstalk at the microbiota-mucosal interface. Proc. Natl. Acad. Sci. U S A. 108 Suppl 1, 4607–4614. doi: 10.1073/pnas.1000092107, PMID: 20826446 PMC3063605

[B117] Wu S. Liu J. Zhang Y. Song J. Zhang Z. Yang Y.. (2022). Structural characterization and antagonistic effect against P-selectin-mediated function of SFF-32, a fucoidan fraction from Sargassum fusiforme. J. Ethnopharmacol. 295, 115408. doi: 10.1016/j.jep.2022.115408, PMID: 35659565

[B118] Xiao L T. R. Wang J. Wan D. Yin Y. Xie L. (2023). Gut microbiota bridges the iron homeostasis and host health. Sci. China Life Sci. Epub 66, 1952–1975. doi: 10.1007/s11427-022-2302-5, PMID: 37515687

[B119] Xie S. Z. Liu B. Ye H. Y. Li Q. M. Pan L. H. Zha X. Q.. (2019). Dendrobium huoshanense polysaccharide regionally regulates intestinal mucosal barrier function and intestinal microbiota in mice. Carbohydr Polym. 206, 149–162. doi: 10.1016/j.carbpol.2018.11.002, PMID: 30553308

[B120] Xu S. Y. Huang X. Cheong K. L. (2017). Recent advances in marine algae polysaccharides: isolation, structure, and activities. Mar. Drugs 15 (12), 388. doi: 10.3390/md15120388, PMID: 29236064 PMC5742848

[B121] Xu X. Zhang X. (2015). Effects of cyclophosphamide on immune system and gut microbiota in mice. Microbiol. Res. 171, 97–106. doi: 10.1016/j.micres.2014.11.002, PMID: 25553830

[B122] Yang C. Merlin D. (2024). Unveiling colitis: A journey through the dextran sodium sulfate-induced model. Inflammatory Bowel Diseases. 30, 844–853. doi: 10.1093/ibd/izad312, PMID: 38280217 PMC11063560

[B123] Yang H. Mirsepasi-Lauridsen H. C. Struve C. Allaire J. M. Sivignon A. Vogl W.. (2020). Ulcerative Colitis-associated E. coli pathobionts potentiate colitis in susceptible hosts. Gut Microbes 12, 1847976. doi: 10.1080/19490976.2020.1847976, PMID: 33258388 PMC7781664

[B124] Yang J. Zhao Y. Shao F. (2015). Non-canonical activation of inflammatory caspases by cytosolic LPS in innate immunity. Curr. Opin. Immunol. 32, 78–83. doi: 10.1016/j.coi.2015.01.007, PMID: 25621708

[B125] Ye Z.-W. Yuan Z.-Y. Wang J. Li H. Li C.-F. Xu G.-H.. (2024). Fucoidan attenuates chronic colitis and behavioral deficits by reshaping gut microbiota-brain axis balance. J. Funct. Foods. 112, 105951. doi: 10.1016/j.jff.2023.105951

[B126] Zahan M. S. Hasan A. Rahman M. H. Meem K. N. Moni A. Hannan M. A.. (2022). Protective effects of fucoidan against kidney diseases: Pharmacological insights and future perspectives. Int. J. Biol. Macromol 209, 2119–2129. doi: 10.1016/j.ijbiomac.2022.04.192, PMID: 35500767

[B127] Zhang M. Li X. Zhang Q. Yang J. Liu G. (2023). Roles of macrophages on ulcerative colitis and colitis-associated colorectal cancer. Front. Immunol. 14, 1103617. doi: 10.3389/fimmu.2023.1103617, PMID: 37006260 PMC10062481

[B128] Zhang X. Yang H. He Y. Zhang D. Lu G. Ren M.. (2025). Yeast-inspired orally-administered nanocomposite scavenges oxidative stress and restores gut immune homeostasis for inflammatory bowel disease treatment. ACS Nano. 19, 7350–7369. doi: 10.1021/acsnano.4c18099, PMID: 39943645

[B129] Zhao X. Di Q. Liu H. Quan J. Ling J. Zhao Z.. (2022). MEF2C promotes M1 macrophage polarization and Th1 responses. Cell Mol. Immunol. 19, 540–553. doi: 10.1038/s41423-022-00841-w, PMID: 35194174 PMC8975968

[B130] Zheng W. Tang S. Ren X. Song S. Ai C. (2024). Fucoidan alleviated colitis aggravated by fiber deficiency through protecting the gut barrier, suppressing the MAPK/NF-κB pathway, and modulating gut microbiota and metabolites. Front. Nutr. 11, 1462584. doi: 10.3389/fnut.2024.1462584, PMID: 39925971 PMC11802440

[B131] Zhou T. Y. Xiang X. W. Du M. Zhang L. F. Cheng N. X. Liu X. L.. (2019). Protective effect of polysaccharides of sea cucumber Acaudina leucoprocta on hydrogen peroxide-induced oxidative injury in RAW264. 7 Cells Int. J. Biol. Macromol 139, 1133–1140. doi: 10.1016/j.ijbiomac.2019.08.092, PMID: 31419551

